# Machine Learning-Based Predictive Model for Tensile and Flexural Strength of 3D-Printed Concrete

**DOI:** 10.3390/ma16114149

**Published:** 2023-06-02

**Authors:** Ammar Ali, Raja Dilawar Riaz, Umair Jalil Malik, Syed Baqar Abbas, Muhammad Usman, Mati Ullah Shah, In-Ho Kim, Asad Hanif, Muhammad Faizan

**Affiliations:** 1 School of Civil and Environmental Engineering (SCEE), National University of Sciences and Technology (NUST), H-12 Sector, Islamabad 44000, Pakistan; 2 School of Electrical Engineering and Computer Science (SEECS), National University of Sciences and Technology (NUST), H-12 Sector, Islamabad 44000, Pakistan; 3 Department of Civil Engineering, Kunsan National University, Kunsan 54150, Republic of Korea; inho.kim@kunsan.ac.kr; 4 Civil and Environmental Engineering Department, King Fahd University of Petroleum and Minerals (KFUPM), Dhahran 31261, Saudi Arabia; 5 Interdisciplinary Research Center for Construction and Building Materials, King Fahd University of Petroleum and Minerals (KFUPM), Dhahran 31261, Saudi Arabia

**Keywords:** 3D-printed concrete, machine learning, additive manufacturing, predictive models, flexural strength, decision tree

## Abstract

The additive manufacturing of concrete, also known as 3D-printed concrete, is produced layer by layer using a 3D printer. The three-dimensional printing of concrete offers several benefits compared to conventional concrete construction, such as reduced labor costs and wastage of materials. It can also be used to build complex structures with high precision and accuracy. However, optimizing the mix design of 3D-printed concrete is challenging, involving numerous factors and extensive hit-and-trail experimentation. This study addresses this issue by developing predictive models, such as the Gaussian Process Regression model, Decision Tree Regression model, Support Vector Machine model, and XGBoost Regression models. The input parameters were water (Kg/m^3^), cement (Kg/m^3^), silica fume (Kg/m^3^), fly ash (Kg/m^3^), coarse aggregate (Kg/m^3^ & mm for diameter), fine aggregate (Kg/m^3^ & mm for diameter), viscosity modifying agent (Kg/m^3^), fibers (Kg/m^3^), fiber properties (mm for diameter and MPa for strength), print speed (mm/sec), and nozzle area (mm^2^), while target properties were the flexural and tensile strength of concrete (MPa data from 25 literature studies were collected. The water/binder ratio used in the dataset ranged from 0.27 to 0.67. Different types of sands and fibers have been used, with fibers having a maximum length of 23 mm. Based upon the Coefficient of Determination (R^2^), Root Mean Square Error (RMSE), Mean Square Error (MSE), and Mean Absolute Error (MAE) for casted and printed concrete, the SVM model performed better than other models. All models’ cast and printed flexural strength values were also correlated. The model’s performance has also been checked on six different mix proportions from the dataset to show its accuracy. It is worth noting that the lack of ML-based predictive models for the flexural and tensile properties of 3D-printed concrete in the literature makes this study a novel innovation in the field. This model could reduce the computational and experimental effort required to formulate the mixed design of printed concrete.

## 1. Introduction

**Figure 1:**
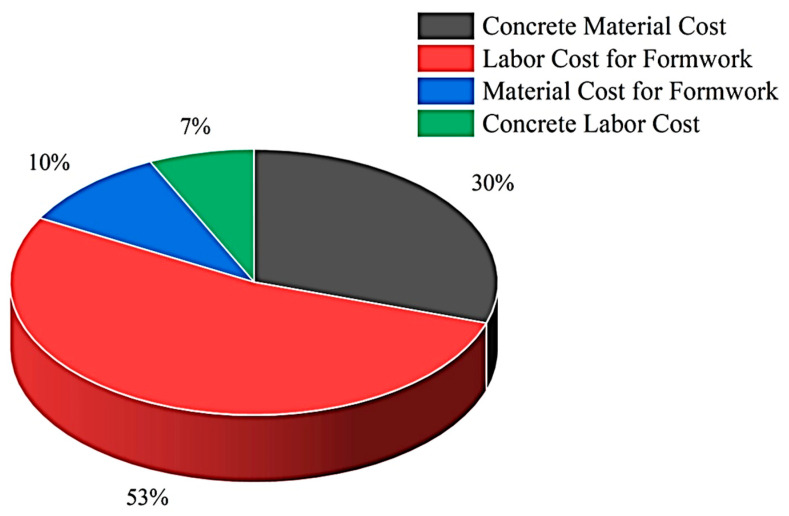
Inclusive distribution of traditional concrete construction expenses. Restated from [46].

Concrete is undoubtedly the most prevalent construction material on earth [1,2,3]. With its versatile applications [4,5,6,7,8], unmatched strength [9,10], and remarkable durability [11,12,13], it has become an integral part of our daily life. From its multifaceted usage in towering skyscrapers [14] and iconic spans [15] to underwater roadways [16] and simple abodes, concrete has become an indispensable factor in shaping the future of humanity [17]. Concrete, an essential construction industry element, is also a cornerstone for the country’s economic development [18,19]. However, new research in civil engineering has transformed this old building material into a powerful and modern construction tool for a sustainable and eco-friendly future [20,21,22,23,24,25,26,27,28,29,30,31,32,33,34,35]. Recently a new way of approaching concrete construction has been developed, which has paved the way for realizing digital construction [36,37,38]. This type of additive manufacturing technology involves layer-by-layer stacking of concrete material in a controlled manner to print the whole structure [39]. It can potentially revolutionize the construction industry by reducing construction costs by 50 to 60% [40]. The conventional way of construction relies upon labor for most of the work. Assembling formwork, preparing, pouring, and demolding concrete is highly time-consuming, laborious, and suspectable to errors [41,42,43]. Formwork accounts for 60% of total construction cost, 10% percent of formwork material, and 50% is labor used to design, install, and remove temporary formwork construction [44]. A detailed distribution of the cost of conventional construction is shown in Figure 1. Three-dimensional (3D) Concrete Printing (3DCP) has the potential to eliminate the role of both formwork and labor, thus reducing the cost of construction. 

This technology has gained significant attention from researchers due to its potential to offer sustainable solutions to building projects. It also provides greater design flexibility as it can create a complex and modular shape with relatively less cost than traditional construction [45].

In terms of modular construction, Ana et al. proposed the replacement of conventionally constructed columns with bespoke columns offering intricate and customizable designs at a lesser cost [47]. Tsinghua University created a prefabricated bridge using 3DCP, with its assembly as compression members. It was printed in prefabricated parts, which were later assembled on-site [48]. Although 3DCP is a growing trend in the construction sector, making a mix suitable for printing remains challenging. The interdependence of machine properties and time-dependent properties of concrete mix makes it a challenging task for optimum printability.

A critical aspect of 3D-printed concrete is selecting appropriate raw materials and mix design that ensures pumpability, extrudability, and buildability to achieve a successful printing process. It should be noted that these characteristics are essential for 3D printing and go beyond the standard workability requirements mentioned in codes and guidelines [49]. The mix design of 3D-printed concrete involves determining the optimal proportions of different materials such as binders, aggregates, chemical admixtures, fibers, and additives to achieve the specific properties required for the final usage. The mix design should also consider the printing method, equipment used, and any design requirements for the final product, such as shape, size, and surface finish. Proper mix design is crucial for successfully producing 3D-printed concrete with the desired properties and characteristics [50].

The properties of the mix design of 3D-printed concrete include workability, rheology, strength, durability, setting time, shrinkage, etc. [50,51,52]. The mixture must have sufficient workability to facilitate precise extrusion, printing, or layering while maintaining the product’s structural sound [53]. The consistency of the mixture must exhibit ideal flow characteristics and viscosity to guarantee effortless extrusion and steadfast adhesion to precede layers while preventing any potential distortion or sagging known as extrudability [45]. The ideal mixture must exhibit compressive and flexural strength that fulfills the structural demands of the end product [54]. It should be tough enough to withstand environmental elements such as moisture, chemical exposure, and freeze-thaw cycles for long-term durability [55]. A suitable setting time is critical to facilitate proper interlayer bonding, prevent printing deformities, and avoid premature cracking [56]. Additionally, the mixture must exhibit minimal shrinkage to avert cracking and distortion over time [57]. While 3D Concrete Printing (3DCP) has many advantages over conventional construction, it also has some unique challenges. One of the potential challenges of 3D Concrete Printing is the development of an innovative mix design to achieve an optimal balance between hardened and fresh-state properties [49]. 

A higher yield stress is favorable for the buildability of concrete in 3D printing; it is, however, essential to ensure that the extrudability of the mix is not compromised, as this parameter is equally important in achieving successful printing [58]. When the yield stress of the concrete is high, it may result in a discontinuous flow of the material through the nozzle during the printing process. This can cause tearing defects in the final printed layer [59]. The literature shows that the addition of cement content, nano clay [60,61], fiber content [62,63,64,65], silica fume [66,67], and fly ash [68,69,70,71] increases the yield strength of the concrete mix for printing purposes. Still, at the same time, they decrease the extrudability of the printable mix. However, the addition of SCMs and cement can increase mechanical strength. Similarly, adding a superplasticizer [60] and water content [72] increases extrudability, but at the same time they decrease the yield stress and mechanical strength of printable concrete. Using an accelerator or rapid-setting cement can increase the buildability of the layer [73,74], but it ultimately affects the long term strength of the concrete. In addition, adding an accelerator can cause pore formation in the concrete. Incorporating fibers into the concrete mix formulated for 3D printing has been found to positively affect its mechanical strength, as per the findings of Panda et al. Nonetheless, this alteration may reduce the workability of the concrete, which can cause complications during the extrusion process [75]. Khalil et al. explored the impact of a viscosity-modifying admixture on the extrudability of limestone. They calcined clay-based cementitious material for 3D concrete printing and found that using such additives can improve printing speeds and surface quality [76], thus increasing the durability of printed elements. The study by Sukontasukkul et al. examines the effect of a viscosity modifier agent (VMA) on the layer deformation, viscosity, and open time of cement mortar for 3D printing applications. The research findings suggest that incorporating VMA into the cement mortar mix can improve printability by increasing viscosity, extending open time, reducing layer deformation, and enhancing the printed structures’ overall quality [77]. The printing speed, nozzle size, and pressure can significantly impact the mechanical properties of 3D-printed concrete filaments. Slow printing speeds can lead to better layer adhesion and increased strength but may not be practical for larger structures due to longer printing times [78]. A larger nozzle size can result in weaker bonding between layers and decreased mechanical properties. In comparison, a smaller nozzle size can improve layer adhesion and mechanical properties but may increase printing time. Pressure affects the flow rate and consistency of the printed material, which can impact the quality and mechanical properties of the printed filament [79,80,81]. 

The conclusion drawn from the studies on 3D concrete printing is that the complex printing process and the multiple factors affecting the mix design make it a challenging and iterative task, requiring extensive experimentation and testing to achieve optimal mix design. Therefore, to address this issue, advanced analytical techniques should be used to develop a mix design for concrete printing, enabling a more efficient and accurate approach towards formulating a mix with suitable rheological and mechanical properties of printed concrete. 

Machine learning is a sub-branch of artificial intelligence that revolves around developing statistical models and algorithms that empowers computers to adaptively learn from antecedent data and evolve without hard coding [82,83]. The importance of using ML-based modeling in civil engineering is advancing incrementally as civil engineering projects and research evolve, becoming more complex and demanding more sophisticated tools and techniques [84,85,86,87]. By creating various mathematical models, the actual behavior of the material is captured, and accurate predictions of different properties, such as failure, strength, durability, deformation, etc., can be obtained [88,89,90,91]. Computational models also allow engineers to simulate different rheological behavior of concrete [92,93,94,95,96]. Engineers and researchers can develop cost-effective and suitable mix designs in this way. 

Similarly, 3D Concrete Printing has attracted research recently because of its potential to revolutionize the construction industry. Much research is being conducted to formulate an appropriate mix design less laboriously. ML modeling is a valuable tool for engineers and professionals involved in 3D printing and design. It can be used to model the different applications of ML in 3D printing, including process optimization, quality control, material development, and design optimization [97]. It can also predict the mechanical properties of printed elements. Jayasudha et al., 2022 employed ANN and Decision Tree Regression to predict the tensile strength of printed elements [98]. Similar findings are obtained in [99,100]. ML modeling can cater to various parameters related to the concrete mix design. It provides an efficient way to optimize the mix design and achieve a given application’s desired strength and durability. The model can be a reliable and accurate solution for the 3D concrete printing industry. 

This study developed different Machine Learning Models, i.e., Gaussian Process Regression, Decision Tree Regression, Support Vector Machine, and XGBoost Regression, to predict the cast and printed anisotropic flexural strength and printed tensile strength of concrete. Data from already studied literature is used. Input parameters include water, cement, silica fume, fly ash, coarse aggregate, fine aggregate, viscosity modifying agent, fibers, fiber properties, print speed, nozzle shape (nozzle area was used to cater to the nozzle shape as there was no consistent data available on the shape), the cast and anisotropic flexural strength of printed concrete, and the tensile strength of printed concrete. Various statistical evaluations such as MSE, RMSE, R^2^, and MAE (MPA) were applied to assess the accuracy of the models to find the most accurate model.

### 1.1. Objectives 

This study aims to investigate the application of different machine learning models to predict concrete’s cast and printed anisotropic flexural strength and printed tensile strength at 28 days. The following are the main objectives of this research.

1. A model was developed to accurately predict concrete’s anisotropic flexural strength and printed concrete’s tensile strength. Additionally, the sensitivity of the models will be analyzed using various statistical approaches.

2. To analyze the most accurate model’s performance using random mix designs of concrete from the dataset.

### 1.2. Research Motivation and Significance

3D Concrete Printing is an advanced technology that enables efficient and sustainable construction. Compared to conventional concrete construction, it has several potential benefits. However, creating a concrete mixture with the proper mechanical and rheological properties requires much experimentation and trial and error [54,101,102,103,104,105,106,107]. Thus, finding a straightforward and less computational approach for developing a mixed design for printable concrete would contribute to overcoming one of the most significant hurdles in implementing this technology in the field. Different researchers have employed machine learning techniques to predict concrete mix properties. A similar research approach has been utilized to optimize the concrete mix design for printing through different machine learning techniques such as Gaussian Process Regression, Decision Tree Regression, Support Vector Machine, and XGBoost Regression.

Various input parameters, such as mix constituent, constituent properties, printer properties, and cement, are taken as independent variables, and the cast and anisotropic flexural strength of printed concrete (MPa) and tensile strength of printed concrete (MPa) are taken as dependent variables. For testing the model, the independent variables were used to calculate the values of the dependent variables. Predicted and original values were compared to evaluate the model.

By examining multiple modeling techniques, this study contributes to developing a novel, accurate, and efficient method for predicting the properties of newly printed concrete structures. This, in turn, can help engineers and researchers optimize the printing process and improve the strength of the final extruded layers. Furthermore, using advanced machine learning algorithms in the optimization process can reduce the computational cost, time, and effort required for accurate predictions, making it a valuable tool for the automated construction industry.

## 2. Methodology

For modelling purposes, different approaches were considered, and their possibles usage in terms of limited dataset was researched i.e., ANNs offers the potential to use multiple hidden layers and non-linear functions to model the output. However, due to the limited dataset samples available, ANNs were not chosen as they rely heavily on the quantity of data samples present. The limited number of data samples would result in overfitting of the ANN to the data present, instead of learning the underlying relations and generalizing well [108]. One-dimensional CNNs are often used for regression tasks when the dataset is time series or sequential, or when a high number of samples are present. As our dataset is not based on time series, and the parameters are not inherently related, this approach was not considered in our empirical analysis. Furthermore, the 1D CNN approach requires thousands of samples for effective modelling, which were not available in our dataset [109]. Autoencoders have the capability to learn a function that maps a set of inputs to a set of outputs by compressing the input into a simplified, compressed code and then reconstructing the input from this code. Autoencoders can be used for regression tasks if the output of the second function is changed from the original input to the target variable. However, due to the limited size of our dataset, autoencoders were not chosen for regression analysis as they require high amounts of data to effectively model the data [110,111].

Therefore, the current study analyzed the structure of a given dataset through statistical analysis. Based on this analysis, multiple regression models were shortlisted. Experiments were conducted to conduct an empirical analysis, on which model would optimally model the relations between the dependent and independent variables. One of the key factors considered while selecting a model was the complexity and non-linearity of the relations between the data. Thus, any model chosen must have the ability to form such functions. Four machine learning algorithms, including Support Vector Regression (SVR), Gaussian Process Regression (GPR), Decision Tree Regression (DTR), and XGBoost, were chosen due to their ability to model non-linear mappings. Furthermore, the parameters available in the data mostly contained continuous nominal data, which made these algorithms well-suited to the data.

Another factor that was considered was the limited availability of data. The number of samples available in the data was the maximum available, so the models SVR, XGBoost, and GPR were chosen. Although Decision Tree Regression requires more extensive data, techniques such as pruning can address this issue. Additionally, the variance in the data was not very high, so even the Decision Tree model was expected to perform well. Lastly, all the models used in this study are flexible in the choice of parameters available. These models offer a range of parameters that can be adjusted to yield optimal results per dataset. Therefore, the models were adjusted based on the available data to achieve the best results. The current study selected four machine learning algorithms well-suited to continuous data that can model complex, non-linear relations between the dependent and independent variables. The study also considered the limited data availability and the models’ flexibility in parameter selection.

**Figure 2:**
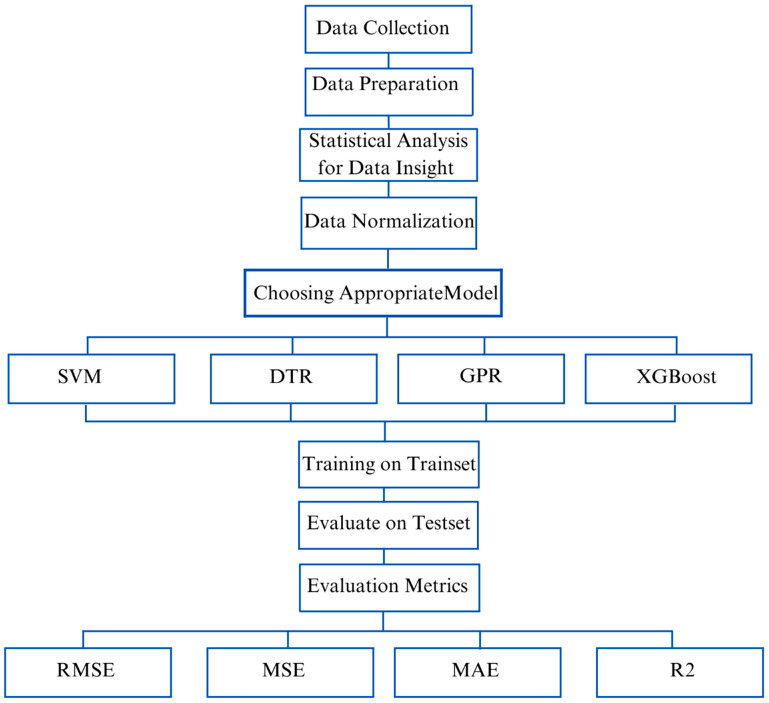
Workflow of research methodology.

**Figure 3:**
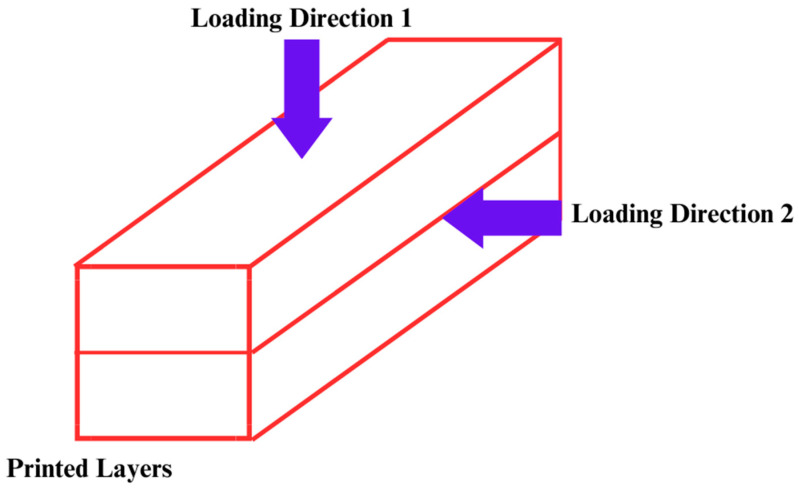
Anisotropic behavior of printed concrete in flexure. Directions are the same as those used in the whole research.

Figure 2 presents a general overview of the whole work methodology used in this research. Two different hardened state properties of printed concrete were studied from the available literature, and reliable datasets were generated. Printed concrete has bi-directional flexural strength [50] (directions 1 and 2 used in the fundamental research are as shown in Figure 3), implying it can resist bending moments in both longitudinal and transverse directions and tensile strength is unidirectional (with testing as referred to in the literature [112]) [113,114]. The limited availability of datasets can be attributed to the emerging scrutiny of this technology. The datasets used in our research methodology can be attributed to the complex nature of the technology, limited adoption, active research, lack of standardization of testing, and privacy infringement. Similarly, this technology places significant emphasis on developing appropriate mix designs. As a result, a vast array of materials is utilized by researchers to investigate and determine optimal mix proportions. 

### 2.1. Regression through Machine Learning Approaches

Over the past decade, machine learning has been used to model real-life problems and successfully assist humanity in handling those [115,116,117]. State-of-the-art development of concrete mixtures and their sophisticated applications have spawned a necessity to use more precise and numerical models to predict their properties. Various researchers have widely used empirical and statistical models in concrete technology [118]. The different kinds of models used by various researchers in concrete research are summarized in Table 1. Our research is focused on developing four different models to predict flexural strength and tensile strength using Decision Tree Regression, Support Vector Machine (SVM) Regressor, Gaussian Process Regressor, and Extreme Gradient Booster Regressor. The detailed overview of machine learning models developed using these algorithms for predicating the flexural and tensile strength can be seen in the Supplementary Materials.

#### 2.1.1. Decision Tree Regressor

Decision Tree Regression is a type of machine learning algorithm that is widely used to partition the input data into smaller subsets. These are widely used for modeling data with the nonlinear or branched relationship between input features and targeted variables. The relationship between input and targeted variables determines the decision rule used to predict future outcomes [119,120]. The Decision Tree Regressor has been used in concrete research to predict various properties of concrete. Karbassi et al., 2014 used this technique to make a quantitative damage prediction tool for regular reinforced concrete [121]. Erdal 2013 used an ensemble of decision trees to predict the compressive strength of concrete [122]. This technique has also been successfully employed to predict carbonation depth in concrete by Taffese et al., 2015 [123]. 

Similarly, other work has also been reported in the literature to predict concrete properties [124,125,126,127]. Decision Tree Regressors can be a powerful technique in 3D concrete printing because of their ability to model complex nonlinear relationships between input variables and the target variable (e.g., compressive strength, flexural strength, and tensile strength of the printed concrete). Three-dimensional concrete printing involves numerous process variables that affect the extruded layers, including the composition of the concrete mixture, nozzle diameter, printing speed, layer thickness, and curing conditions, so the Decision Tree Regressor technique can be promising in accurately predicting final layer properties.

#### 2.1.2. Support Vector Machine Regressor

Support Vector Machine Regression is a highly supervised and classical machine-learning regression modeling and analysis algorithm. It tries to fit the best possible line, thus producing continuous output on new input data [128]. This highly supervised and classical machine-learning algorithm technique relies on the statistical learning methodology to generate, train, and optimize models [129]. Support Vector Machine (SVM) regression involves mapping the input data x to a high-dimensional feature space through nonlinear mapping, followed by linear regression in this space. The regression model is expressed as y = f(x) + e, where x and y are input and output functions defined in the high-dimensional feature space, and e is an independent random error. The regression function f(x) is also defined in the feature space, allowing for nonlinear regression in the original input space [130]. This approach enables SVM regression to accurately model complex non-linear relationships between input and output variables. Because of the ability of support vector machines to handle high-dimensional data, robustness to data noise, and higher generalized performance have been widely used to analyze complex chemical spectra and analysis of compounds [131]. This has been widely used in concrete research because of its accurate insensitive loss function. Yan et al., 2010 showed that SVM performed well and outperformed other models in predicting the elastic modulus of concrete that involves elaborate testing under cyclic loading and strain measurement [132]. Sonebi et al., 2016 used radial basis function (RBF) and polynomial kernels to predict the fresh properties of self-compacting concrete as a function of the content of mix components [133]. Abd et al., 2017 found SVM to be a valuable tool for predicting the compressive strength of lightweight foamed concrete with minimal mean square errors and standard deviation [134]. Conventional proportioning methods suffer from high costs, usage constraints, and an inability to capture the intricate nonlinear relationships between concrete properties and constituent components. SVM, as the alternative method, was found by Mohtasham Moein et al., 2023 to be imperative to address these limitations and provide a more efficient and effective way of proportioning concrete mixtures. Gupta 2007 utilized 190 dataset points by experimental investigation in the laboratory and made an SVM model to predict 28 days of compressive strength of concrete with a correlation coefficient of 0.996 [135].

The 3D printing of concrete is a rapidly evolving technology that has the potential to revolutionize the construction industry. However, the complex properties of 3D-printed concrete present significant challenges in predicting the performance of the final structure. Support Vector Machines (SVM) have emerged as a powerful tool for predicting the complex properties of 3D-printed concrete. SVM can handle large datasets with high-dimensional inputs, making it suitable for modeling the intricate relationships between the input parameters and the output properties of 3D-printed concrete. Moreover, SVM can effectively handle the nonlinear relationships between the input and output variables, which is standard in 3D printing processes.

**Table 1:** Different models used by various researchers to predict the properties of different types of concrete along with performance criteria.

Authors	Techniques	Data Sources	Performance Metrics	Predictions	References
Chang et al., 2022	U-net convolutional neural network is used to predict the cracking pattern in printed concrete, then by taking the crack pattern as input, Principal Component Analysis (PCA) and CNN are used for the prediction of stress crack width curves.	Binary Image of Air Void Structure through X CT and Fracture Analysis of printed concrete.	R squared value of 0.8.	Predicted the stress-crack curves of printed concrete.	[136]
Charrier et al., 2022	Artificial Neural Networking is employed to predict dynamic yield stress and mini-slump of concrete based on mix proportions affecting the rheology of concrete.	Experimental data from mini-slump cone test and dynamic yield stress through rheometer.	R squared value of 0.93.	Predicted dynamic yield stress and mini-slump of concrete.	[137]
Izadgoshasb et al., 2021	Multi-objective grasshopper optimization algorithm (MOGOA) and artificial neural network (ANN) are employed for the prediction of the compressive strength of printed concrete.	Experimental dataset through literature.	The correlation coefficient of 0.96.	Predicted compressive strength of printed concrete.	[138]
Czarnecki et al., 2021	Artificial Neural Network (ANN), Support Vector Machine, and random forest algorithm are used to predict interlayer bonding.	Conducted research and measurements in the literature.	The correlation coefficient of 0.883.	Predicted interlayer bonding/pull-off adhesion of concrete layers.	[139]
Zhang et al., 2019	Long Short-term Memory (LSTM) network is used with Fused Deposition Modelling (FDM) to predict the tensile strength of printed concrete.	Data from experimental runs.	The correlation coefficient of 0.773.	Predicted tensile strength of printed concrete.	[140]
Bagheri et al., 2020	Conditional inference tree (ctree) and recursive partitioning (rpart) functions are used to predict the compressive strength of geopolymers.	Experimental data of boron-based geopolymer concrete from literature studies.	70% cumulative accuracy.	Predicted compressive strength of printed geopolymer concrete.	[141]
Lao et al., 2021	Artificial Neural Networking is used to predict the final geometry of concrete filament.	Pretesting setup with different nozzle shapes and extruded filaments.	Reduction of 38% in the mean arithmetic roughness (*R_t_*).	The predicted final geometry of extruded layer of printed concrete.	[142]

#### 2.1.3. Gaussian Process Regressor

A non-probabilistic and non-parametric machine learning technique is often used for regression analysis. It differs from Decision Tree Regression and SVM because it does not assume a unique functional form to carry out the modeling of the dataset. Rather, it models the distribution of the dataset directly. Gaussian Process Regressor (GPR) is a probabilistic machine learning technique that uses Bayesian inference to make predictions based on the observed data. Given a training set *D* = {(*xi*, *yi*) | *i* = 1, …, *n*}, GPR assumes that the output variable 𝑦 is a function of the input variable 𝑥, which can be modeled as a Gaussian Process. The Gaussian Process is fully specified by a mean function (*x*) and a covariance function *K*(*x*, *x*′), which are used to estimate the conditional probability distribution of the output variable 𝑦 given the input variable 𝑥 [143]. The design matrix *X* is used to define the input space of the Gaussian Process, and the vector of desired output 𝑦 is used to train the model. The primary assumption of GPR is that the output 𝑦 is computed as 𝑦 = 𝑓(𝑥) + ε, where *f*(*x*) is the unknown proper function and ε is the additive Gaussian noise with mean zero and variance σ2. GPR assumes that (*x*) follows a Gaussian Process, and therefore the predicted output *y*∗ for a new input *x*∗ is also a Gaussian distribution [144]. Various researchers have used this technique to predict concrete properties. Dutta et al., 2018 predicted the compressive strength of concrete using GPR. According to Słoński’s findings in 2011, the benchmark dataset revealed that Bayesian neural networks and Gaussian processes have comparable prediction accuracy and outperform the linear regression model [145]. In the study by Omidinasab et al. (2022), the comparative performance of different models in predicting the shear strength of reinforced concrete was analyzed. The results showed that the Gaussian process regression model outperformed the other models, with an R^2^ coefficient of 0.91 and the lowest error [146]. According to the findings of Kovačević et al., 2021, the Gaussian Process Regression (GPR) model with significantly lower complexity had accuracy criterion values comparable to those of the most accurate model. In addition, it was demonstrated that feature reduction could be easily incorporated into GPR using Automatic Relevance Determination (ARD), resulting in models that exhibit better performance and lower complexity [147].

Gaussian Process Regression (GPR) can be beneficial in predicting the properties of printed concrete because it is a robust machine learning algorithm that can effectively capture the complex and non-linear relationships between the input and output parameters. GPR models can provide accurate predictions, even with limited data and noisy measurements, which makes them a valuable tool for predicting the properties of printed concrete, i.e., flexural, tensile, and compressive strength, etc.

#### 2.1.4. Extreme Gradient Booster Regressor (XG-Booster)

Extreme Gradient Booster Regression is a robust machine-learning algorithm that is used for regression tasks. XGBoost Regression is an ensemble method that utilizes the strength of multiple decision trees to make accurate predictions [148]. Due to its superior performance, XGBoost is widely recognized as a highly effective machine-learning algorithm capable of handling large datasets with remarkable speed and precision. Its ability to handle complex relationships between input and output variables makes it a popular choice for various applications [149]. The XGBoost algorithm is known for its ability to handle sparse data and implement distributed and parallel computing flexibly, making it a popular choice for solving machine learning and data mining problems. With its powerful computing capabilities, XGBoost has emerged as a promising tool for various applications in the field of data sciences [150] The Nguyen et al. (2021) study employs four predictive algorithms to predict high-performance concrete’s compressive and tensile strengths. The models, including Support Vector Regression (SVR), Multilayer Perceptron (MLP), Gradient Boosting Regressor (GBR), and Extreme Gradient Boosting (XGBoost), are trained using a hyperparameter tuning process based on a random search. The missing data is handled by filling it with the mean of the available data to maximize information utilization in the training process. The results showed that the GBR and XGBoost models outperform the SVR and MLP models in terms of both prediction accuracy and computational efficiency [151].

XGBoost is a robust machine learning algorithm that has the competency to handle larger datasets and complex features, which means it can be an efficient tool in handling the properties of printed concrete. By leveraging machine learning models such as Extreme Gradient Booster Regressor (XGBoost), engineers and researchers can accurately predict the properties of newly printed concrete structures. This allows for optimizing the printing process, as engineers can adjust various parameters and settings based on the predicted properties to achieve desired performance characteristics. This can result in significant cost reduction and enhanced performance for 3D Concrete Printing in the construction industry.

### 2.2. Overview of Dataset

**Figure 4:**
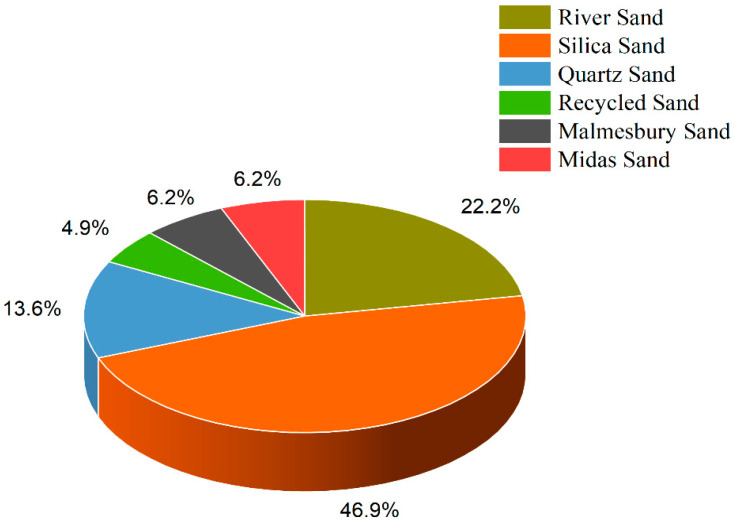
Pie Chart showing data distribution of sand types used in modeling. This is done to cater to the effect of different sand types on the properties of concrete, considering their maximum sizes.

**Figure 5:**
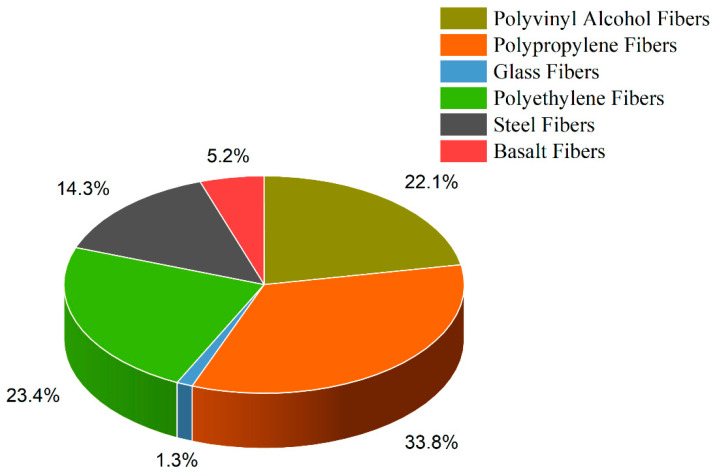
Pie Chart showing data for types of fibers used in modeling while catering the effect of fiber size, diameter, Young’s modulus, and tensile strength.

This study comprehensively analyzed 77 mix designs to generate a model for flexural strength [44,107,152,153,154,155,156,157,158,159,160,161,162,163,164,165,166,167]. Additionally, 49 mix designs were examined to develop a model for tensile strength [117,118,119,120,121,122,129,132,133,134,135]. The data collected from these mix designs were used to train and test the models to predict novel mix designs’ flexural and tensile strengths accurately. The dataset incorporates water, ordinary Portland cement, silica fume, fly ash, nano clay, Viscosity Modifying Agent (VMA), and coarse aggregate quantified with a maximum size of 10mm fine aggregate, classified based on a maximum size of 0.9mm and type of sand used. The mix design chosen for inclusion in the dataset is fiber reinforced. The quantity, type, tensile strength, Young’s modulus, length, and fiber diameter have been accurately quantified. In the context of the mechanical properties of the printer, the linear printing speed of the nozzle and nozzle correctional area have been included in the dataset to provide valuable insights into the printing process and the generalized effect of the mechanical aspect of printing technology. Figure 4 and Figure 5 shows pie chart distributions of the sand fibers used in the dataset.

### 2.3. Details of Dataset

#### 2.3.1. Cement

Cement can have both positive and negative effects on the properties of concrete. Higher cement dosage can increase early age strength but also cause higher heat of hydration and autogenous and drying shrinkage cracks [168,169,170,171].

#### 2.3.2. Fibers

Adding fibers to 3D Printing Concrete (3DPC) mixtures can improve the mechanical and physical properties of the printed parts. The fiber reinforcement can increase the tensile and flexural strength, toughness, and crack resistance of 3DPC. However, the choice of fiber type, content, and distribution must be carefully considered to avoid potential adverse effects on printability and workability [112,170]. 

#### 2.3.3. Fine Aggregate

Fine aggregate is crucial in 3D Printing Concrete mixtures to ensure strength and stability. However, the particle size and shape of the fine aggregate impact rheological properties and printability, with smoother and more spherical particles improving flowability and lowering the viscosity [105,172]. A higher percentage of sand can stiffen the material and negatively affect extrudability and printability, highlighting the need to carefully optimize sand content and properties [169].

#### 2.3.4. Coarse Aggregate

Coarse aggregate can positively impact 3D Printing Concrete by enhancing its mechanical properties, reducing shrinkage, and decreasing costs. However, the aggregate’s size and shape can affect the mix’s workability and extrudability, and larger particles may cause clogging in the printing nozzle [173]. Therefore, carefully selecting and optimizing the aggregate size and shape are crucial to ensure optimal performance in 3D Printing Concrete.

#### 2.3.5. Fly Ash

Fly ash, a by-product of coal-fired power plants, can partially replace cement in 3D Printing Concrete (3DPC) mixtures. Fly ash can improve the workability, printability, and mechanical properties of 3DPC while reducing the environmental impact of concrete production [174].

#### 2.3.6. Silica Fume

Silica fume can be used as an additive in 3D Printing Concrete mixtures to improve its properties, such as increasing compressive strength, reducing drying shrinkage, and improving durability. It can also reduce the heat of hydration and mitigate the risk of thermal cracking. However, its use may require adjustments to the mix design and printing parameters [67,175].

#### 2.3.7. Superplasticizer 

Superplasticizers can be used in 3D Printing Concrete to improve workability, increase flowability, and reduce viscosity, resulting in better extrudability and printing performance. They can also improve the strength and durability of the final product by reducing the water-to-cement ratio and increasing the compactness of the concrete matrix. However, excessive use of superplasticizers can cause segregation and bleeding, reducing homogeneity and structural integrity [176,177]. Proper dosage and selection of superplasticizers are therefore critical for achieving the desired properties in 3D Printing Concrete.

#### 2.3.8. Accelerator

Accelerators are chemical additives used in 3D Printing Concrete mixtures to adjust working performance and achieve desired properties, such as increasing early age strength and decreasing setting time value. However, excessive use of accelerators can cause a rapid increase in the heat of hydration, leading to thermal cracks [73,74].

### 2.4. Statistical Analysis of Data

Statistical insight was obtained to understand and interpret datasets—the output of X train. describe () provided a helpful starting point for exploring and understanding the training data and helped select appropriate data preprocessing techniques and machine learning models. The range, mean, and standard deviation for the features of the dataset for flexural and tensile strength are shown below in Table 2.

**Figure 6:**
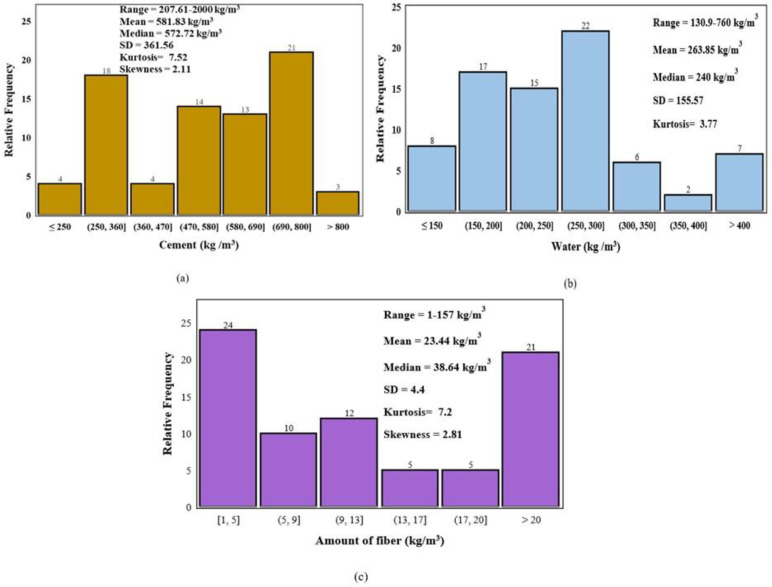
Relative frequency distribution of (**a**) Cement, (**b**) Water, and (**c**) Fiber in Kg/m^3^ for Flexural Strength data.

**Figure 7:**
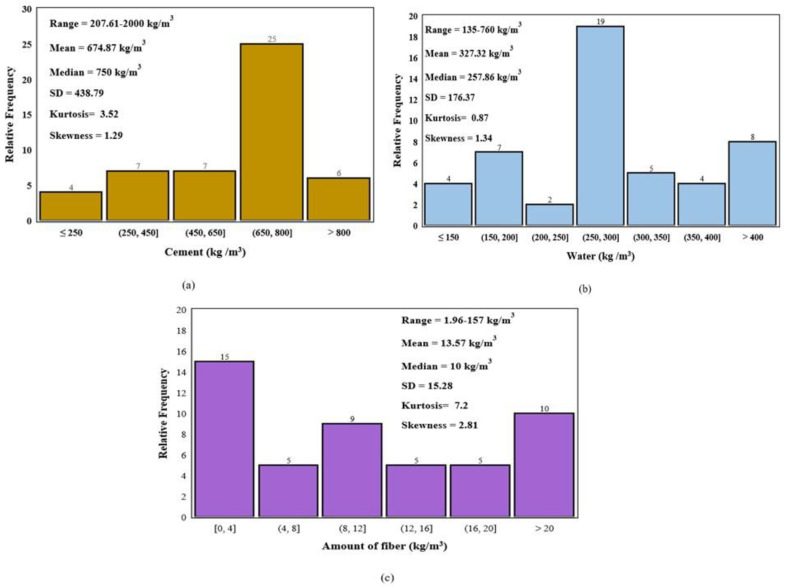
Relative frequency distribution of (**a**) Cement, (**b**) Water, and (**c**) Fiber in Kg/m^3^ for Tensile Strength data.

Relative frequency graphs showing the percentage occurrence of different features in the dataset are shown below in Figure 6 and Figure 7 for flexural and tensile strength of printed concrete. By analyzing these graphs, the visual distribution and relative frequency of each feature can be observed.

#### 2.4.1. Data Cleaning

In this step, the datasets for both properties were analyzed for identification, correction, and removal of inconsistencies. The mean value was calculated and filled in place for the missing datasets for Print Speed, Max Size, and Nozzle Area.

#### 2.4.2. Data Normalization

To improve the accuracy of the models, the datasets in both models were subjected to data normalization. In this process, the numerical features of our dataset were scaled using Min–Max scaling. For data normalization, Equation (1) was used.

(1) $$x^{*}=\frac{x-x_{\min}}{x_{\min}-x_{\max}}$$

Here, x* is the normalized value of the parameter, *x* is the original value, *x*(min) is the lowest value of that parameter, and *x*(max) is the highest value of the parameter.

From the dataset, various parameters, such as water content (Kg/m^3^), cement content (Kg/m^3^), silica fume content (Kg/m^3^), fly ash content (Kg/m^3^), coarse aggregate (Kg/m^3^ & mm diameter), fine aggregate (Kg/m^3^ & mm diameter), viscosity modifier (Kg/m^3^), fibers (Kg/m^3^), fiber properties (mm diameter and MPa strength), print speed (mm/sec), and nozzle area (mm^2^) are taken as independent variables. The cast and anisotropic flexural strength of printed concrete (MPa) and the tensile strength of printed concrete (MPa) are dependent variables. For testing each model, the independent variables were used to calculate the values of the dependent variables. To evaluate each model, the original and predicted values were compared.

### 2.5. Evaluation Criteria

Two distinct sets of evaluation criteria have been established in evaluating the accuracy of the regression models. In a recent study, mixtures were assessed based on their flexural strength, when cast and printed in both directions 1 and 2, and their tensile strength. The study utilized 57 mix designs for training the model for flexural strength, which was subsequently evaluated on 20 additional mix designs in a 3:1 ratio. To evaluate tensile strength, 35 mix designs were used to train the model, and 14 were used for testing. 

#### 2.5.1. Mean Square Error

The regression model evaluation is performed by measuring the average squared magnitude of errors generated by the models. A higher value of Mean Squared Error (MSE) indicates that the model’s predictions are, on average, less accurate, with a larger average squared magnitude of errors between the predicted values and the actual values of the target variable.

(2) $$MSE=\frac{1}{n}{\sum_{i=1}^{n}\left(Y_{pre}-Y_{actual}\right)}^{2}$$

#### 2.5.2. Coefficient of Determination: (R-Squared/R^2^)

Regressions models were also evaluated based on the statistical measure of the portion of variations in the dependent variable predicted from the independent variable(s) through regression models. The values of R^2^ should lie between 0 and 1. A value of 1 indicates that all of the variations in the dependent variable can be explained by the independent variable(s). In contrast, a value of 0 indicates that none of the variations in the dependent variable can be explained by the independent variable(s). A value between 0 and 1 indicates the proportion of the variance in the dependent variable that can be explained by the independent variable(s). The formula to calculate the R^2^ value in terms of the predicted value *Y_pre_* and the actual value *Y_act_* is as stated below:

(3) $$R^{2}=\frac{\sum_{i=1}^{n}\left(Y_{pre}-\bar{Y_{pre}}\right)}{\sum_{i=1}^{n}\left(Y_{actual}-\bar{Y_{actual}}\right)}$$

#### 2.5.3. Mean Absolute Error: (MAE) 

MAE stands for Mean Absolute Error, a commonly used metric in regression analysis to measure the accuracy of a regression model’s predictions. MAE calculates the average difference between the predicted and actual values of the dependent variable. The absolute value ensures that the errors are positive and ignores the direction of the error.

A lower MAE value indicates that the model’s predictions are more accurate, while a higher MAE value suggests that the model’s predictions are less accurate. MAE is useful for comparing the performance of different regression models and selecting the one with the lowest MAE value.

(4) $$MAE=\frac{\sum_{i=1}^{n}\left|y_{i}-x_{i}\right|}{n}$$

where y_i_ is the predicted value, x_i_ is the actual value, and n is the total number of datasets.

#### 2.5.4. Root Mean Square Error: (RMSE)

Root Mean Square Error (RMSE) is a commonly used metric to measure the difference between predicted and actual values in statistical analysis and machine learning. It is the square root of the average squared differences between predicted and actual values. RMSE measures the accuracy of a model’s predictions, with lower values indicating better accuracy. It is a helpful metric for evaluating regression models and is commonly used in economics, engineering, and physics.

The formula to calculate Root Mean Square Error is as follows:

(5) $$MSE=\sqrt{\frac{1}{n}{\sum_{i=1}^{n}\left(Y_{pre}-Y_{actual}\right)}^{2}}$$

From the sensitivity analysis, it can be seen that the influence of data with larger values is not present in the models. From Table 2, it can be seen that cement, water, fly ash, and coarse aggregate amounts are larger compared to other parameters. From sensitivity analysis, it can be seen that the flexural strength is most sensitive to the amount of fibers (Kg/m^3^) and tensile strength is most sensitive to the tensile strength of fibers (MPa). 

### 2.6. Hyperparameter Tuning

Hyperparameter tuning is finding the best combination of hyperparameters for a machine-learning algorithm to achieve the best performance. The settings that affect the behavior and performance of an algorithm cannot be learned from data. Hyperparameter tuning involves exploring different values for these hyperparameters and selecting the optimal values based on evaluating the model’s performance on a validation set. Optimizing hyperparameters can significantly enhance a model’s accuracy and ability to generalize. It is an important stage in building machine learning models and is imperative to attain cutting-edge outcomes. 

In this step, optimal values of hyperparameters were found to achieve the best possible performance of the linear regression model, shown in Table 3 and Table 4. For the case of multiple training, the optimal values are bolded.

## 3. Results and Discussion 

Based on the hyperparameters in the table, the models were trained and evaluated for the two sections below. 

### 3.1. Predicted Results and Discussions

Using datasets for both, flexural and tensile models were trained for all techniques and their accuracy was quantified in terms of Mean Square Error and Coefficient of Determination. The closing value of the Mean Square Error to zero means that the accuracy of the employed model is better. The value of the coefficient of correlation is from 0 to 1. A value of 1 for R^2^ means that the model perfectly predicted the target variable and a value of zero indicates the model does not explain the variance in the dataset. In general, a higher R^2^ value indicates that the model is better at explaining the variation in the target variable. The obtained performance results exhibit a high level of excellence and are deemed suitable for predictive purposes, surpassing the results of previous research studies. The figures below provide a graphical representation of the detailed comparison between the actual and predicted outcomes.

#### 3.1.1. Decision Tree Regressor

**Figure 8:**
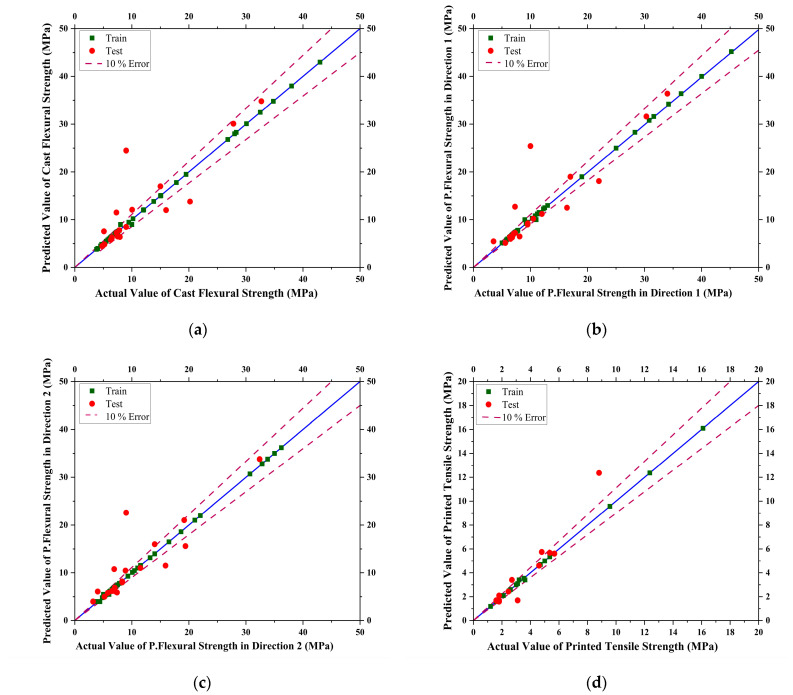
Curves showing actual vs. predicted results for Decision Tree Regression model for (**a**) Casted Flexural Strength, (**b**) Printed Flexural Strength in Direction 1, (**c**) Printed Flexural Strength in Direction 2, (**d**) Printed Tensile Strength of concrete.

For the case of Decision Tree Regression, the default parameters criterion = ‘squared_error’, splitter = ‘best’, max_depth = None, min_samples_split = 2, and min_samples_leaf = 1 were applied for the decision tree model, which was used to evaluate the flexural strength and tensile strength of mix designs in the study. For the flexural strength evaluation, the model was trained on an input dataset, using the default squared_error criterion to measure the quality of the splits in the decision tree. The best splitter strategy was used to choose the best split among all possible splits, and the default max_depth parameter allowed the tree to expand until all leaves were pure or until all leaves contain less than min_samples_split samples, which is set to 2 by default. The min_samples_leaf parameter was also set to the default value of 1, which sets the minimum number of samples required to be at a leaf node. The same strategy was applied to the tensile strength modeling. The trained values and tested values with 10% error lines for data scattering are shown below in Figure 8.

#### 3.1.2. Support Vector Machine 

The SVM model was used to predict the flexural and tensile strength of mixtures based on their composition. The kernel function was used, which determines the shape of the decision boundary used to separate the different classes in the regression problem. The three kernel functions used are the linear, Radial Basis Function (RBF), and sigmoid kernels.

**Figure 9:**
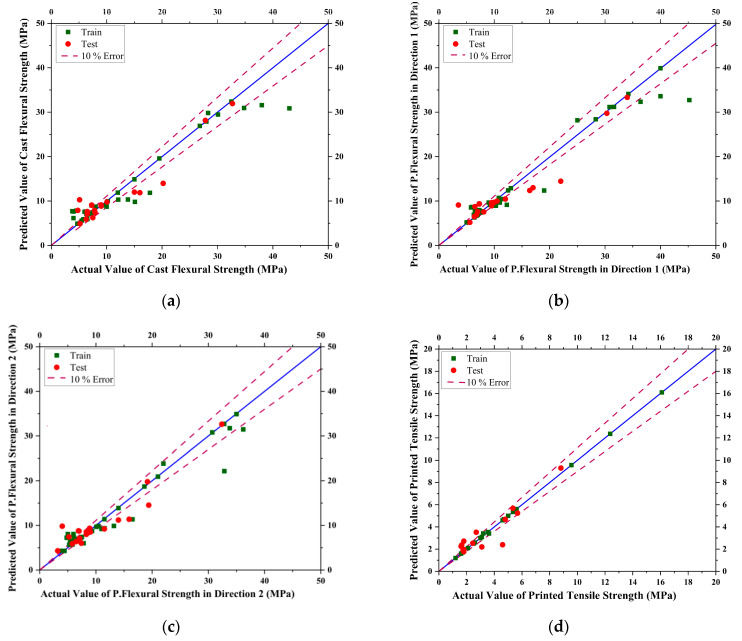
Curves showing actual vs. predicted results for Support Vector Machine (SVM) model for (**a**) Casted Flexural Strength, (**b**) Printed Flexural Strength in Direction 1, (**c**) Printed Flexural Strength in Direction 2, (**d**) Printed Tensile Strength of concrete.

The models were trained and evaluated on five levels of degree in modeling. The degree of the polynomial kernel determines the complexity of the decision boundary, with higher degrees allowing for more complex decision boundaries. The degrees used were 2, 3, 4, 5, and 7. The optimum degree for the polynomial kernel function was found in terms of the evaluation criterion established. The trained values and tested values with 10% error lines for data scattering are shown below in Figure 9.

#### 3.1.3. Gaussian Process Regressor

**Figure 10:**
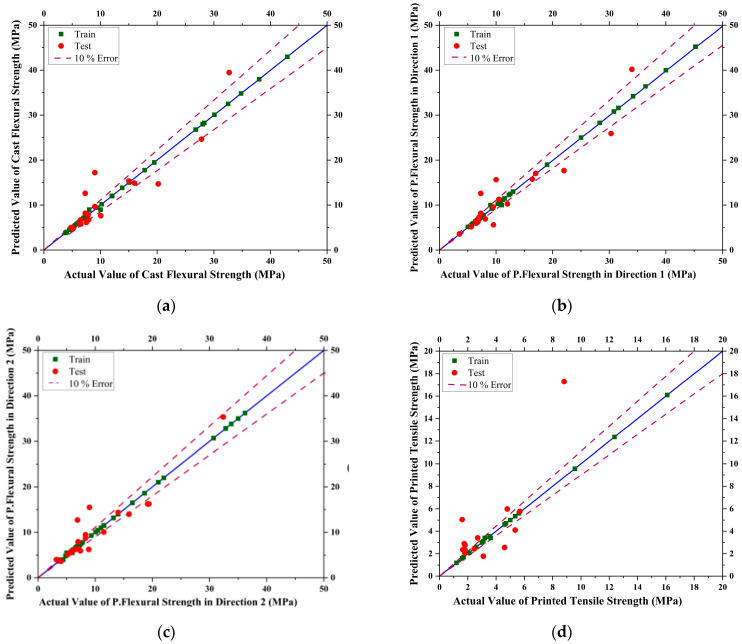
Curves showing actual vs. predicted results for Gaussian Process Regressor (GPR) model for (**a**) Casted Flexural Strength, (**b**) Printed Flexural Strength in Direction 1, (**c**) Printed Flexural Strength in Direction 2, (**d**) Printed Tensile Strength of Concrete.

The models were trained and tested in Gaussian Process Regressor for the given dataset. The trained values and tested values with 10% error lines for data scattering are shown below in Figure 10.

#### 3.1.4. XGBOOST Regressor

**Figure 11:**
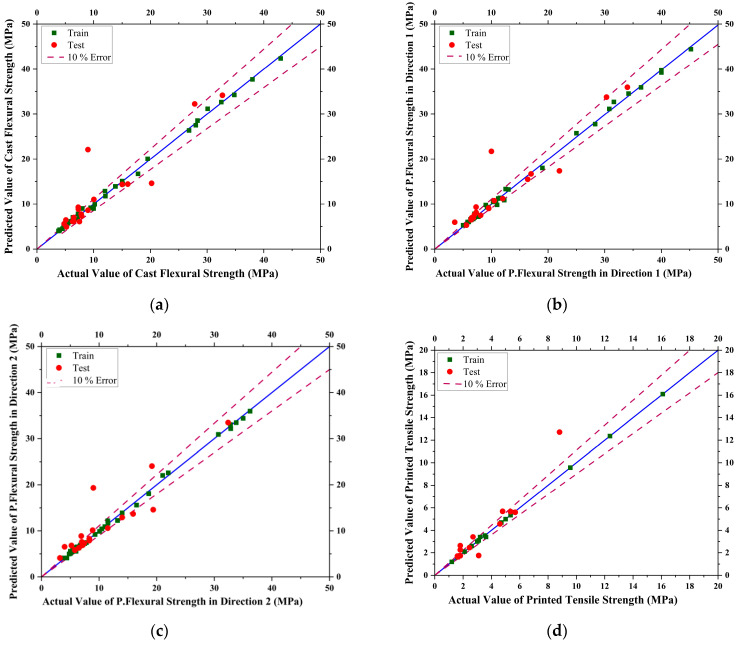
Curves showing actual vs. predicted results for XGBoost Process Regressor for (**a**) Casted Flexural Strength, (**b**) Printed Flexural Strength in Direction 1, (**c**) Printed Flexural Strength in Direction 2, (**d**) Printed Tensile Strength of concrete.

The gradient boosting regression model was developed in scikit-learn python library. This model was used to predict the flexural and tensile strength of mixtures based on their composition. The model was optimized during training using the mean squared error loss function, with a learning rate of 0.1. The model consists of 100 trees, with each tree fitting on a subset of the data defined by the subsample parameter, which has a default value of 1.0. The quality of each split in the decision tree was evaluated using the Friedman mean squared error criterion, which has a default value of ‘friedman_mse’. The min_samples_split parameter was used to control the minimum number of samples required to split an internal node, with a default value of 2. To assess the accuracy of the trained model, a subset of the available data was used for testing, with the remaining data used for training. In this context, the flexural strength model was trained and evaluated on datasets, and the tensile strength model was also trained. The trained values and tested values with 10% error lines for data scattering are shown below in Figure 11.

The research compares different types of machine learning algorithms and their difference between predicted results and actual values. The table in the hyperparameter tuning section assesses the effect of changing hyperparameters on the results of the model and contrasts between the R^2^ and RMSE of different algorithms. 

The best results were generated with the SVM algorithm, i.e., R^2^ and RMSE. Different hyperparameters for SVM were used for empirical analysis to fine-tune the results. Among that, the best results for tensile were on a linear kernel (R^2^ of 0.8454), MAE of 0.6108, MSE of 0.6603 MPa, and RMSE of 0.8126, while for flexural they were with a poly kernel of degree 5 (R^2^ of 0.9009, 0.8936, and 0.8785); MAE of 1.5843, 1.689, and 1.6301; MSE of 5.5837 MPa, 6.9089 MPa, and 5.59 MPa; and RMSE of 2.3629 MPa, 2.6284 MPa, and 2.3643 MPa on Casted, Direction 1, and Direction 2, respectively. SVM can handle high-dimensionality data and model the complex non-linear relationships among it.

The Decision Tree Regressor and XG-Boost Regressor can model relations between discrete data or non-linear relations between attributes. However, both these algorithms bear a high risk of overfitting. The Decision Tree Regressor and XGBoost regressor generate optimal results on the training data; however, these fail to generalize and provide unsatisfactory results on the test set. Both have low R^2^ scores and high RMSE values. For tensile, the R^2^ for the Decision Tree Regressor is 0.72036 and 0.67230 for the XGBoost regressor, and RMSE is 1.09311 MPa for the Decision Tree Regressor and 1.18332 for the XGBoost regressor, which is worse than all experiments with SVM. 

For flexural, the Gaussian Process Regressor had results comparable to the best, with R^2^ of 0.8265, 0.87778, and 0.8673 and RMSE of 3.1262 MPa, 2.8174 MPa, and 2.4701 MPa for Casted, Direction 1, and Direction 2. In contrast, it had the worst results among all models on the tensile dataset, with an R^2^ of -0.61268 and RMSE of 2.62509 MPa. This could be because the Gaussian Process Regressor is non-parametric and learns essentially from data, which was deficient for tensile but not flexural.

### 3.2. Sensitivity Evaluation

The most influential mix constituent on the cast and printed anisotropic flexural and printed tensile strength of 3D-printed concrete was identified using sensitivity analysis [178]. The process involved removing one input parameter at a time and calculating the MAE and RMSE for each trial. The sensitivity of the input parameters was ranked, and the mix constituent that had the most significant impact on the concrete’s strength properties was determined. This approach helped to optimize the concrete’s properties for 3D printing purposes.

The results of the sensitivity analysis for flexural strength are presented in Table 5.

### 3.3. Validation of Predictive Models

**Figure 12:**
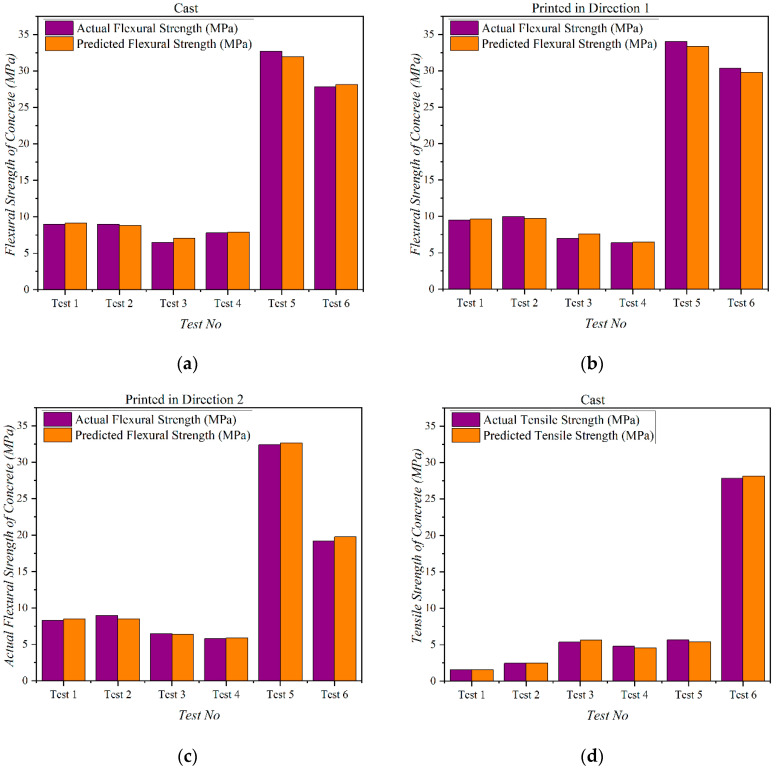
Comparison of predicted results for (**a**) Casted Flexural Strength, (**b**) Printed Flexural Strength in Direction 1, (**c**) Printed Flexural Strength in Direction 2, (**d**) Printed Tensile Strength of concrete.

Although the model’s performance was outstanding when processing the comprehensive data used for training, it was imperative to evaluate its accuracy on entirely new data that could be either part of the dataset or not. Consequently, six mix designs were selected from the dataset to conduct the validation. These are shown in Table 6 and Table 7 below. The prediction results are summarized in Figure 12 and indicate that the model’s accuracy evaluation criteria were better. However, available literature indicates no prior instance of this model being trained on 3D concrete printing. The findings demonstrate that the model’s performance is still robust and dependable, even when tested on novel data from the dataset. 

## 4. Conclusions

ML-based predictive models for the flexural and tensile strength of 3D-printed concrete do not exist in the literature. Therefore, this paper aimed to develop an accurate ML-based predictive model for concrete’s cast and printed anisotropic flexural and printed tensile properties. For this purpose, the data was collected from the literature and used to train, validate, and test four different predictive models based on the ML techniques Decision Tree Regression, Support Vector Machine (SVM) Regressor, Gaussian Process Regressor, and Extreme Gradient Booster Regressor. The primary research outcomes are as follows:

Based on the collected data from the literature, the Support Vector Machine Regression-based predictive model presents the highest degree of accuracy compared to the Decision Tree Regressor, Gaussian Process Regressor, and Extreme Gradient Booster Regressor. 

For the case of printing in Direction 1, the Coefficient of Relation (R^2^_score) for SVM is 0.8936, while for DTR, GPR, and XGBOOS, it is 0.7253, 0.8997, and 0.8571, respectively. Similarly, for printing in Direction 2, the Coefficient of Relation (R^2^_score) for SVM is 0.8785, while for DTR, GPR, and XGBOOS, it is 0.7166, 0.8919, and 0.8237, respectively. The highest value of R^2^ of SVM compared to other techniques indicates better data fitting to the regression model.

The values of R^2^ reported in this research are comparable to the findings by reporting values of 0.84, 0.94, 0.945, and 0.92.

Although the dataset used in the study is limited in availability, the MAE, RMSE, and MSE values also indicate a better performance of developed models, as indicated by the test mix.

Similarly, as indicated in Table 3 and Table 4, the lowest RMSE, MSE, and MAE value for SVM indicated less deviation of predicted values from the actual values.

As indicated by the sensitivity analysis performed in Table 3 and Table 4, the most influential parameter on casted and printed flexural strength of concrete is the number of fibers (Kg/m^3^) in the mix design. For tensile strength, it is the tensile strength of fibers (MPa) used in the mix design, although these are not the parameters with highest data in the complete dataset. This shows that parameters with the largest data do not affect the accuracy of the models.

Trail mixes from the dataset with variable compositions are used for evaluating the models. The mean error for casted flexural strength is ±1.2 MPa, for printed flexural strength in Direction 1 it is ±1.3 MPa, for printed flexural strength in Direction 2 it is ±1.2 MPa, and for printed tensile strength it is ±0.26 MPa. 

The least accurate predictive models for the tensile and flexural strength of 3D-printed concrete are based on the Gaussian Process Regressor and Decision Tree Regressor, respectively.

The outcome of this research is an accurate predictive model that can be used to predict the cast and printed anisotropic flexural strength and printed tensile strength of concrete. Based on the evaluation criteria RMSE, MSE, R^2^, MAE, and Sensitivity Analysis, the Support Vector Machine (SVM) Regression Model yields the most accurate result. The findings provide a basis for using such techniques for practical implementation to overcome the rigorous and challenging iterative task of mix design formulation of 3D-printed concrete.

In our study, one of the major challenges encountered was the small sample size, which posed a risk of overfitting the model during training. We evaluated multiple regression methods such as the Decision Tree Regressor, Extreme Gradient Boosting Regressor, Gaussian Process Regressor, and Support Vector Regressor (SVM Regressor). However, we observed that the methods Decision Tree Regressor and Extreme Gradient Boosting Regressor tended to overfit due to their reliance on the dataset, as they failed to learn the underlying functions or distributions to generalize well. While GPR works by fitting a Gaussian distribution to the data, it is prone to overfitting due to its flexibility in adjusting parameters to fit closely to the data, particularly when the amount of data was limited. Furthermore, GPR could capture noise in the data, which contributed to overfitting. However, we found that the SVM Regressor performed better than other methods in our dataset due to its ability to transform data into higher order planes through kernel transformations and to find a function for the relationships between dependent and independent variables. This allows the SVM Regressor to generalize well, even with limited sample size, as it focuses on learning the underlying relationships rather than the data itself. Moreover, the regularization term in the SVM regressor helps to lower the risk of overfitting. Additionally, our dataset had a high number of parameters, which further enhanced the performance of the SVM regressor, as it could create a higher-dimensional space to capture the relationships between different parameters. Furthermore, unlike GPR, the SVM regressor was less prone to outliers and noise in the data, as it relied more on support vectors, and outliers had less weight in the overall model.

Therefore, we conclude that the SVM regressor is a more suitable method for regression analysis when dealing with limited sample size, a high number of parameters, and the presence of outliers and noise in the data.

In conclusion, the research has successfully developed a predictive model using machine learning that can accurately estimate the flexural and tensile strength of 3D-printed concrete. This model can be highly beneficial to the construction industry as it enables the efficient selection of optimal ingredients without time-consuming laboratory trials. Moreover, the model’s accurate predictions can lead to the improved structural integrity of 3D-printed concrete structures, which is critical for ensuring the safety and longevity of such constructions. Overall, the machine learning-based predictive model developed in this study has the potential to revolutionize the construction industry by enabling efficient and cost-effective production of 3D-printed concrete structures.

### 4.1. Limitation and Scope of the Study

Due to the limited available dataset, this research could not utilize the potential of deep learning, in general, and neural networks, specifically. One of the limitations of this study is the number of available datasets. Three-dimensional concrete printing is an emerging construction technology worldwide, and it is currently being researched so that the available dataset is limited and could be much better in the future. This technology needs universal standardization of testing. Similarly, most printer designs are different, and modeling is quite complex. Still, to overcome the issues of artificiality induced by fewer datasets, the data points were carefully selected from 25 studies from the available literature. Then, advanced machine-learning approaches, such as Gaussian Process Regression, Support Vector Machine Regression, Decision Tree Regression, and XGBoost Regression, were used. The model’s accuracy was evaluated based on the Coefficient of Correlation, Mean Absolute Error (MAE), Root Mean Square Error (RMSE), and Mean Square Error (MSE).

### 4.2. Feasibility of Work and Potential Impact

Making a concrete mix with suitable rheological and mechanical properties is quite an extensive and challenging task. Therefore, the approach researched in the paper provides a feasible solution for an individual researcher or engineer to predict and achieve the optimal mechanical parameters of printable concrete. 

This research on concrete mix design using ML modeling to predict the mechanical strength (flexural and tensile strength) of printable concrete can make the 3D concrete printing of structures faster and less expensive. This is because it will reduce the need for trial and error, leading to more accurate predictions of the mechanical strength of the concrete.

### 4.3. Future Work

While this research yielded remarkable results, there exists potential for future work. The current dataset needed to be more extensive in the number of samples available and might experience the problem of overfitting. A new research dimension is to augment the data through multiple techniques and compare the results on different deep learning architectures. Neural networks can model complex non-linear relationships and learn the patterns between data hidden and overlooked by traditional machine learning algorithms. To counter this problem, a rudimentary approach is to collect more data samples, while an advanced approach is to apply data augmentation techniques. Data augmentation techniques such as Generative Adversarial Networks (GANs) for tabular data have produced considerably realistic datasets that could be used for training and testing.

## Figures and Tables

**Table 2:** Statistical analysis of data shown by the range, mean, and standard deviation for the features of the dataset for flexural and tensile strength of the dataset.

Parameters	Units	Range		Mean		Standard Deviation	
		Flexural Data	Tensile Data	Flexural Data	Tensile Data	Flexural Data	Tensile Data
**Cement**	Kg/m^3^	207.61–2000	207.61–2000	581.83	674.87	361.57	438.79
**Water**	Kg/m^3^	130.9–760	135–760	263.85	327.32	155.58	176.37
**Silica Fume**	Kg/m^3^	0–293	0–280	82.09	55.94	97.94	91.51
**Fly ash**	Kg/m^3^	0–1380	0–1380	272.84	321.27	345.58	446.78
**HRWA**	Kg/m^3^	0–36	0–36	6.39	5.68	7.53	8.79
**Accelerator**	Kg/m^3^	0–146.4	0–146.4	4.5	2.99	19.84	20.91
**Nano Clay**	Kg/m^3^	0–5.89	0–5.89	0.6	0.51	1.58	1.51
**VMA**	Kg/m^3^	0–38.64	0–31.74	2.39	3.78	6.15	10.41
**Coarse Aggregate Amount**	Kg/m^3^	0–1566.3	0–1566.3	189.72	241.16	392.25	494.08
**Coarse Aggregate Size**	mm	0–10	0–10	1.75	1.62	2.84	3.37
**Fine Aggregate**	Kg/m^3^	195.7–3420	195.7–3420	838.48	836.09	593.23	721.23
**Fine Aggregate Size**	mm	0.176–3	0.2–3	0.45	0.53	0.2	0.42
**Tensile Strength of Fibers**	MPa	300–3000	300–3000	1775.8	1564.4	997.5	1087.31
**Young’s Modulus of Fibers**	GPa	3–200	3–300	68.48	71.42	67.94	61.48
**Amount of Fibers**	Kg/m^3^	1–157	1.96–157	23.44	13.57	38.64	15.28
**Length of Fibers**	mm	6–23	6–23	10.47	10.35	4.66	5.36
**Diameter of Fibers**	µm	11.2–200	11.2–300	47.3	31.59	56.05	38.5
**Print Speed**	mm/sec	10–450	10–120	94.22	63.72	109.68	31.55
**Nozzle Area**	mm^2^	50.25–1259	78.54–1256	546.58	580.16	365.87	351.18

**Table 3:** Hyperparameters for the modeling of the tensile strength dataset.

Tensile Strength						
Model	Hyperparameter		Test			
			Direction 1			
			R^2^_Score	RMSE (MPa)	MSE (MPa)	MAE (MPa)
**Decision Tree Regressor**	**Default parameters:** criterion = ‘squared_error’, splitter = ‘best’, max_depth = None, min_samples_split = 2, min_samples_leaf = 1		0.7217	1.0904	1.1889	0.5857
**XGBoost Regressor**	**Default parameters:** loss = ‘squared_error’, learning_rate = 0.1, n_estimators = 100, subsample = 1.0, criterion = ‘friedman_mse’, min_samples_split = 2		0.6757	1.1771	1.3856	0.6255
**Gaussian Process Regressor**	**Default parameters/NA**		−0.8156	2.7853	7.7583	1.7688
**SVM Regressor**	**Parameters:** Kernel = “linear”		**0.8893**	**0.6877**	**0.4729**	**0.5168**
	Kernel = “rbf”		0.7363	1.0614	1.1265	0.7923
	Kernel = “sigmoid”		0.4781	1.4933	2.2299	1.0734
	Kernel = “poly”	degree = 2	0.8697	0.7461	0.5567	0.5619
		degree = 3	0.8705	0.7436	0.5529	0.5294
		degree = 4	0.8726	0.7377	0.5443	0.5230
		degree = 5	0.8454	0.8126	0.6603	0.6108
		degree = 7	0.5856	1.3306	1.7705	0.9655

**Table 4:** Hyperparameters for the modeling of flexural strength.

Flexural Strength														
Model	Hyperparameter		Test											
			Casted				Direction 1				Direction 2			
			R^2^_Score	RMSE (MPa)	MSE (MPa)	MAE (MPa)	R^2^	RMSE (MPa)	MSE (MPa)	MAE (MPa)	R^2^	RMSE (MPa)	MSE (MPa)	MAE (MPa)
**Decision Tree Regressor**	**Default parameters:** criterion = ‘squared_error’, splitter = ‘best’, max_depth = None, min_samples_split = 2, min_samples_leaf = 1		0.7107	4.0378	16.3038	2.1685	0.7253	4.2234	17.8378	2.1450	0.7166	3.6106	13.0370	1.9234
**XGBoost Regressor**	**Default parameters:** loss = ‘squared_error’, learning_rate = 0.1, n_estimators = 100, subsample = 1.0, criterion = ‘friedman_mse’, min_samples_split = 2		0.7826	3.4995	12.2471	1.9138	0.8571	3.0464	9.2805	1.6562	0.8237	2.8478	8.1100	1.6802
**Gaussian Process Regressor**	**Default parameters/NA**		0.8586	2.8223	7.9658	1.7799	0.8997	2.5521	6.5136	1.6653	0.8919	2.2298	4.9724	1.5179
**SVM Regressor**	**Parameters:** Kernel = “linear”		0.8389	3.0131	9.0789	2.2241	0.8302	3.3203	11.0248	2.3213	0.8588	2.5482	6.4936	1.8319
	Kernel = “rbf”		0.3747	5.9361	35.2376	3.4569	0.3441	6.5263	42.5938	3.7748	0.4109	5.2057	27.0992	3.1575
	Kernel = “sigmoid”		0.2753	6.3902	40.8355	3.8144	0.2507	6.9757	48.6608	4.2864	0.2992	5.6780	32.2397	3.5497
	Kernel = “poly”	degree = 2	0.8015	3.3440	11.1820	2.3023	0.7940	3.6570	13.3741	2.4048	0.8556	2.5772	6.6422	1.8418
		degree = 3	0.8798	2.6020	6.7705	1.8348	0.8681	2.9266	8.5651	1.9164	0.8650	2.4914	6.2070	1.7467
		degree = 4	0.8947	2.4357	5.9326	1.5969	0.8824	2.7630	7.6342	1.7180	0.8705	2.4408	5.9577	1.6728
		**degree = 5**	**0.9009**	**2.3629**	**5.5837**	**1.5843**	**0.8936**	**2.6284**	**6.9089**	**1.6899**	**0.8785**	**2.3643**	**5.5900**	**1.6301**
		degree = 7	0.8597	2.8119	7.9068	1.8643	0.8621	2.9921	8.9529	2.0122	0.8713	2.4331	5.9202	1.7106

**Table 5:** Sensitivity analysis of data used for modelling of most accurate regression technique deduced from the research (SVM).

Serial No	Removed Parameter	RMSE (MPa)	MAE (MPa)	Rank	Removed Parameter	RMSE (MPa)	MAE (MPa)	Rank	Removed Parameter	RMSE (MPa)	MAE (MPa)	Rank
	Direction 1				Direction 2				Tensile			
1	water	3.435388	1.707343	16	water	3.054693	1.611342	15	water	1.235907	0.542882	15
2	OPC	3.469017	1.720952	14	OPC	3.08612	1.6242	13	OPC	1.313908	0.58144	6
3	SF	3.679582	1.746922	8	SF	3.295288	1.651021	8	SF	1.372479	0.585067	5
4	FA	3.427675	1.724601	12	FA	3.043816	1.632707	9	FA	1.214197	0.527205	19
5	HRWA	3.49679	1.72722	11	HRWA	3.112101	1.632399	10	HRWA	1.279392	0.555373	12
6	Accelerator	3.429991	1.694391	17	Accelerator	3.051652	1.586162	18	Accelerator	1.242211	0.53891	17
7	Nano Clay	3.443021	1.744391	9	Nano Clay	3.031044	1.608555	16	Nano Clay	1.27908	0.586401	4
8	VMA	3.620481	1.789529	5	VMA	3.233733	1.701543	4	VMA	1.248234	0.550853	13
9	Coarse Aggregate	3.419646	1.687259	19	Coarse Aggregate	3.040884	1.590642	17	Coarse Aggregate	1.227094	0.529152	18
10	Coarse Aggregate Size	3.420119	1.722704	13	Coarse Aggregate Size	3.043928	1.628047	11	Coarse Aggregate Size	1.300266	0.555383	11
11	Fine aggregate	3.467813	1.719678	15	Fine aggregate	3.089325	1.627822	12	Fine aggregate	1.279348	0.561863	7
12	Fine aggregate Max size	3.529115	1.814253	3	Fine aggregate Max size	3.135855	1.678854	7	Fine aggregate Max size	1.286223	0.561064	8
13	Fiber tensile strength	3.578673	1.768906	7	Fiber tensile strength	3.151401	1.705591	3	Fiber tensile strength	1.471981	0.66846	1
14	Fiber Young’s modulus	3.783561	1.84142	2	Fiber Young’s modulus	3.330147	1.711165	2	Fiber Young’s modulus	1.611737	0.660326	2
15	Fiber Amount	3.870431	1.901596	1	Fiber Amount	3.327988	1.717442	1	Fiber Amount	1.287742	0.555659	9
16	Fiber Length	3.620481	1.789529	6	Fiber Length	3.233733	1.701543	5	Fiber Length	1.313256	0.592416	3
17	Fiber Diameter	3.757303	1.796675	4	Fiber Diameter	3.351271	1.701478	6	Fiber Diameter	1.241695	0.539999	16
18	Print speed	3.445291	1.690833	18	Print speed	3.05888	1.585716	19	Print speed	1.199372	0.555412	10
19	Nozzle area	3.495971	1.741203	10	Nozzle area	3.101378	1.623677	14	Nozzle area	1.220588	0.544233	14
20	Fibers	3.232545	1.559722	21	Fibers	2.898589	1.436404	21	Fibers	1.118979	0.510727	20
21	Sand type	3.322575	1.668542	20	Sand type	2.988024	1.514551	20	Sand type	1.102163	0.503668	21

**Table 6:** Mix composition of test mixes for tensile strength model with reported printed tensile strength of concrete.

Tensile Strength Test Mix					
Mix	1	2	3	4	5
**Water (kg/m^3^)**	289.7	256	329.8	177.12	135.19
**Cement (kg/m^3^)**	783	562	565.37	656	207.61
**SF (kg/m^3^)**	39.15	81.4	0	246	0
**FA (kg/m^3^)**	140.9	162	671.38	118	275.21
**HRWA (kg/m^3^)**	0.98	4.8	14.13	3	5.79
**Nano Clay/Nano Clay**	0	0	5.89	0	2.414
**VMA (kg/m^3^)**	0.49	2.41	1.18	0	0.48
**Max Size (mm)**	10	0	0	0	0
**Amount Fine Aggregate (kg/m^3^)**	978.7	1144	471.14	455	275.21
**Max Size (mm)**	0.39	0.4	0.3	0.31	0.2
**Sand Type**	River Sand	Malmmesbury	Silica Sand	Silica Sand	Silica Sand
**Fiber**	PP	PP	PE	PE	PE
**Tensile Strength MPa**	300	300	300	2900	3000
**Young’s Modulus (GPA)**	3	3	116	116	116
**Amount (kg/m^3^)**	1.96	22	40	10	14.55
**Length (mm)**	12	6	12	23	12
**Diameter (micrometer)**	130	30	24	25	24
**Print Speed mm/sec**	100	60	100	10	100
**Nozzle Area mm^2^**	1256	490.625	314	1000	314

**Table 7:** Mix composition of test mixes for flexural strength model with reported cast and printed anisotropic flexural strength of concrete.

Flexure Strength Test Mix						
Mix	1	2	3	4	5	6
**Water (kg/m^3^)**	244	285	210	240	156	154
**Cement (kg/m^3^)**	376	289	350	622	273	750
**SF (kg/m^3^)**	41.36	145	100	88.9	293	165
**FA (kg/m^3^)**	100	277	185	257	0	0
**HRWA (kg/m^3^)**	5.64	9	8	2.57	18	10
**Nano Clay/Nano Clay**	0	0	0	0	5	0
**VMA (kg/m^3^)**	0	0	4	0	0	1.08
**Coarse Aggregate Amount (kg/m^3^)**	0	0	0	0	390	180
**Size**	0	0	0	0	4.75	4.75
**Amount Fine Aggregate (kg/m^3^)**	732.6	1209	750	1066.75	878	924
**Max Size (mm)**	0.4	0.47	0.8	0.38	0.176	0.85
**Sand Type**	Silica Sand	Malmesbury	Midas sand	River Sand	Silica Sand	Quartz Sand
**Fiber**	PVA	Glass	PP	PP	Steel	Steel
**Tensile Strength MPa**	1600	450	300	3000	2500	2500
**Young’s Modulus (GPA)**	30	74	3.85	3	200	200
**Amount (kg/m^3^)**	7	13.5	12.7	1.2	157	39
**Length (mm)**	18	6	6	9	6	10
**Diameter (micrometer)**	39	40	30	23	200	0.12
**Print Speed mm/sec**	110	150	0	450	30	15
**Nozzle Area mm^2^**	112.32	50.25	625	240	706.5	176.625
**Casted**	9	9	6.5	7.8	32.7	27.81
**Direction 1 (MPa)**	9.5	10	7	6.4	34	30.32
**Direction 2 (MPa)**	8.3	9	6.5	5.8	32.4	19.17

## Data Availability

The data used in this research paper will be made available upon request. To ensure the privacy and confidentiality of the data, access will be provided following a reasonable request and upon agreement with the data sharing and usage policies. Interested parties may contact Ammar Ali at aali1.bece19nice@student.nust.edu.pk to inquire about accessing the data used in this study.

## Supplementary Materials

The following supporting information can be downloaded at: https://www.mdpi.com/article/10.3390/ma16114149/s1, contains the supplementary files showing the generalized overview of the modelling procedure used in this research with DOI’s of the data set used.
