# Supplementary material for: Machine Learning-Based Predictive Model for Tensile and Flexural Strength of 3D-Printed Concrete
Source: Materials (Basel). 2023 Jun 2;16(11):4149. doi: 10.3390/ma16114149 (PMC10254869; doi:10.3390/ma16114149)
Supplement: Supplementary file 1 [file materials-16-04149-s001.zip › materials-2353812-supplementary.pdf]

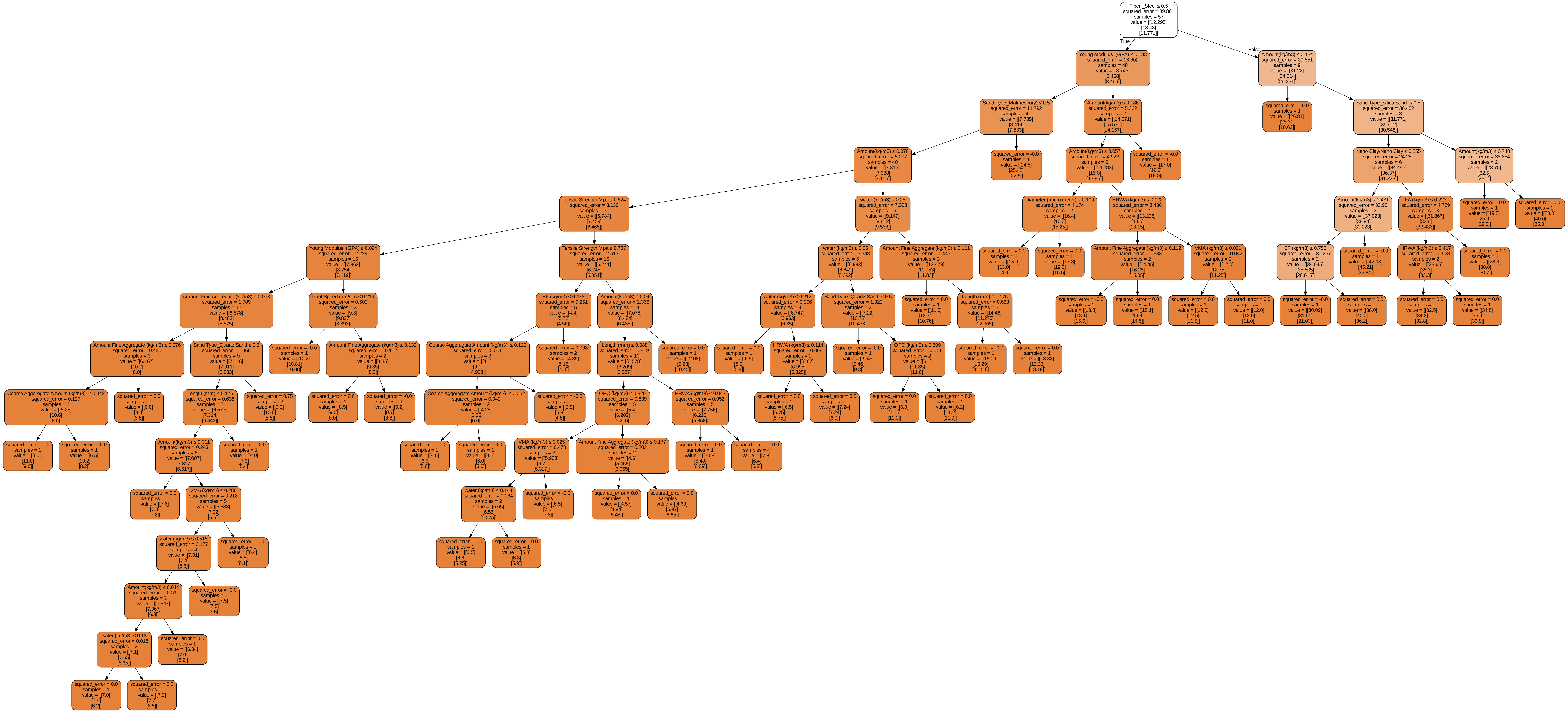
Fiber _Steel ≤ 0.5 squared_error = 89.861 samples = 57 value = [[12.295] [13.43] [11.771]]
True
False
Young Modulus (GPA) ≤ 0.533 squared_error = 16.802 samples = 48 value = [[8.746] [9.458] [8.499]]
Amount(kg/m3) ≤ 0.184 squared_error = 39.551 samples = 9 value = [[31.22] [34.614] [29.221]]
Sand Type_Malmesbury) ≤ 0.5 squared_error = 11.792 samples = 41 value = [[7.735] [8.414] [7.533]]
Amount(kg/m3) ≤ 0.186 squared_error = 5.362 samples = 7 value = [[14.671] [15.571] [14.157]]
squared_error = 0.0 samples = 1 value = [[26.81] [28.31] [18.62]]
Sand Type_Silica Sand ≤ 0.5 squared_error = 36.452 samples = 8 value = [[31.771] [35.402] [30.546]]
Amount(kg/m3) ≤ 0.078 squared_error = 5.277 samples = 40 value = [[7.315] [7.988] [7.156]]
squared_error = -0.0 samples = 1 value = [[24.5] [25.42] [22.6]]
Amount(kg/m3) ≤ 0.057 squared_error = 4.922 samples = 6 value = [[14.283] [15.0] [13.85]]
squared_error = -0.0 samples = 1 value = [[17.0] [19.0] [16.0]]
Nano Clay/Nano Clay ≤ 0.255 squared_error = 24.251 samples = 6 value = [[34.445] [36.37] [31.228]]
Amount(kg/m3) ≤ 0.748 squared_error = 38.854 samples = 2 value = [[23.75] [32.5] [28.5]]
Tensile Strength Mpa ≤ 0.524 squared_error = 3.136 samples = 31 value = [[6.784] [7.459] [6.465]]
water (kg/m3) ≤ 0.28 squared_error = 7.338 samples = 9 value = [[9.147] [9.812] [9.538]]
Diameter (micro meter) ≤ 0.109 squared_error = 4.174 samples = 2 value = [[16.4] [16.0] [15.25]]
HRWA (kg/m3) ≤ 0.122 squared_error = 3.436 samples = 4 value = [[13.225] [14.5] [13.15]]
Amount(kg/m3) ≤ 0.431 squared_error = 33.96 samples = 3 value = [[37.023] [38.94] [30.023]]
FA (kg/m3) ≤ 0.223 squared_error = 4.739 samples = 3 value = [[31.867] [33.8] [32.433]]
squared_error = 0.0 samples = 1 value = [[19.5] [25.0] [22.0]]
squared_error = 0.0 samples = 1 value = [[28.0] [40.0] [35.0]]
Young Modulus (GPA) ≤ 0.094 squared_error = 2.224 samples = 15 value = [[7.363] [8.754] [7.119]]
Tensile Strength Mpa ≤ 0.737 squared_error = 2.513 samples = 16 value = [[6.241] [6.245] [5.851]]
water (kg/m3) ≤ 0.25 squared_error = 3.348 samples = 6 value = [[6.983] [8.842] [8.392]]
Amount Fine Aggregate (kg/m3) ≤ 0.111 squared_error = 1.447 samples = 3 value = [[13.473] [11.753] [11.83]]
squared_error = 0.0 samples = 1 value = [[15.0] [13.0] [14.0]]
squared_error = 0.0 samples = 1 value = [[17.8] [19.0] [16.5]]
Amount Fine Aggregate (kg/m3) ≤ 0.112 squared_error = 1.383 samples = 2 value = [[14.45] [16.25] [15.05]]
VMA (kg/m3) ≤ 0.021 squared_error = 0.042 samples = 2 value = [[12.0] [12.75] [11.25]]
SF (kg/m3) ≤ 0.752 squared_error = 30.257 samples = 2 value = [[34.045] [35.805] [28.615]]
squared_error = -0.0 samples = 1 value = [[42.98] [45.21] [32.84]]
HRWA (kg/m3) ≤ 0.417 squared_error = 0.928 samples = 2 value = [[33.65] [35.3] [33.3]]
squared_error = 0.0 samples = 1 value = [[28.3] [30.8] [30.7]]
Amount Fine Aggregate (kg/m3) ≤ 0.093 squared_error = 1.789 samples = 12 value = [[6.878] [8.483] [6.675]]
Print Speed mm/sec ≤ 0.219 squared_error = 0.602 samples = 3 value = [[9.3] [9.837] [8.893]]
SF (kg/m3) ≤ 0.478 squared_error = 0.251 samples = 5 value = [[4.4] [5.72] [4.56]]
Amount(kg/m3) ≤ 0.04 squared_error = 2.366 samples = 11 value = [[7.078] [6.484] [6.438]]
water (kg/m3) ≤ 0.212 squared_error = 0.206 samples = 3 value = [[6.747] [6.963] [6.35]]
Sand Type_Quartz Sand ≤ 0.5 squared_error = 1.322 samples = 3 value = [[7.22] [10.72] [10.433]]
squared_error = 0.0 samples = 1 value = [[11.5] [12.71] [10.76]]
Length (mm) ≤ 0.176 squared_error = 0.683 samples = 2 value = [[14.46] [11.275] [12.365]]
squared_error = -0.0 samples = 1 value = [[13.8] [18.1] [15.6]]
squared_error = 0.0 samples = 1 value = [[15.1] [14.4] [14.5]]
squared_error = 0.0 samples = 1 value = [[12.0] [12.5] [11.5]]
squared_error = 0.0 samples = 1 value = [[12.0] [13.0] [11.0]]
squared_error = -0.0 samples = 1 value = [[30.09] [31.61] [21.03]]
squared_error = 0.0 samples = 1 value = [[38.0] [40.0] [36.2]]
squared_error = 0.0 samples = 1 value = [[32.5] [34.2] [32.8]]
squared_error = 0.0 samples = 1 value = [[34.8] [36.4] [33.8]]
Amount Fine Aggregate (kg/m3) ≤ 0.076 squared_error = 0.436 samples = 3 value = [[6.167] [10.2] [8.0]]
Sand Type_Quartz Sand ≤ 0.5 squared_error = 1.468 samples = 9 value = [[7.116] [7.911] [6.233]]
squared_error = -0.0 samples = 1 value = [[10.2] [10.81] [10.08]]
Amount Fine Aggregate (kg/m3) ≤ 0.139 squared_error = 0.112 samples = 2 value = [[8.85] [9.35] [8.3]]
Coarse Aggeregate Amount (kg/m3) ≤ 0.129 squared_error = 0.061 samples = 3 value = [[4.1] [6.1] [4.933]]
squared_error = 0.068 samples = 2 value = [[4.85] [5.15] [4.0]]
Length (mm) ≤ 0.088 squared_error = 0.819 samples = 10 value = [[6.578] [6.209] [6.037]]
squared_error = 0.0 samples = 1 value = [[12.08] [9.23] [10.45]]
squared_error = 0.0 samples = 1 value = [[6.5] [6.9] [5.4]]
HRWA (kg/m3) ≤ 0.114 squared_error = 0.068 samples = 2 value = [[6.87] [6.995] [6.825]]
squared_error = -0.0 samples = 1 value = [[9.46] [9.46] [9.3]]
OPC (kg/m3) ≤ 0.309 squared_error = 0.011 samples = 2 value = [[6.1] [11.35] [11.0]]
squared_error = -0.0 samples = 1 value = [[15.09] [10.29] [11.54]]
squared_error = 0.0 samples = 1 value = [[13.83] [12.26] [13.19]]
Coarse Aggeregate Amount (kg/m3) ≤ 0.442 squared_error = 0.127 samples = 2 value = [[6.25] [10.6] [8.6]]
squared_error = 0.0 samples = 1 value = [[6.0] [9.4] [6.8]]
Length (mm) ≤ 0.176 squared_error = 0.638 samples = 7 value = [[6.577] [7.314] [6.443]]
squared_error = 0.75 samples = 2 value = [[9.0] [10.0] [5.5]]
squared_error = 0.0 samples = 1 value = [[8.5] [9.0] [8.0]]
squared_error = -0.0 samples = 1 value = [[9.2] [9.7] [8.6]]
Coarse Aggeregate Amount (kg/m3) ≤ 0.062 squared_error = 0.042 samples = 2 value = [[4.25] [6.25] [5.0]]
squared_error = -0.0 samples = 1 value = [[3.8] [5.8] [4.8]]
OPC (kg/m3) ≤ 0.329 squared_error = 0.639 samples = 5 value = [[5.4] [6.202] [6.216]]
HRWA (kg/m3) ≤ 0.043 squared_error = 0.052 samples = 5 value = [[7.756] [6.216] [5.858]]
squared_error = 0.0 samples = 1 value = [[6.5] [6.75] [6.75]]
squared_error = 0.0 samples = 1 value = [[7.24] [7.24] [6.9]]
squared_error = 0.0 samples = 1 value = [[6.0] [11.5] [11.0]]
squared_error = 0.0 samples = 1 value = [[6.2] [11.2] [11.0]]
squared_error = 0.0 samples = 1 value = [[6.0] [11.0] [9.0]]
squared_error = -0.0 samples = 1 value = [[6.5] [10.2] [8.2]]
Amount(kg/m3) ≤ 0.011 squared_error = 0.243 samples = 6 value = [[7.007] [7.317] [6.617]]
squared_error = 0.0 samples = 1 value = [[4.0] [7.3] [5.4]]
squared_error = 0.0 samples = 1 value = [[4.0] [6.5] [5.0]]
squared_error = 0.0 samples = 1 value = [[4.5] [6.0] [5.0]]
VMA (kg/m3) ≤ 0.025 squared_error = 0.478 samples = 3 value = [[5.933] [6.7] [6.317]]
Amount Fine Aggregate (kg/m3) ≤ 0.177 squared_error = 0.203 samples = 2 value = [[4.6] [5.455] [6.065]]
squared_error = 0.0 samples = 1 value = [[7.58] [5.48] [6.09]]
squared_error = -0.0 samples = 4 value = [[7.8] [6.4] [5.8]]
squared_error = 0.0 samples = 1 value = [[7.6] [7.8] [7.2]]
VMA (kg/m3) ≤ 0.286 squared_error = 0.218 samples = 5 value = [[6.888] [7.22] [6.5]]
water (kg/m3) ≤ 0.144 squared_error = 0.064 samples = 2 value = [[5.65] [6.55] [5.575]]
squared_error = -0.0 samples = 1 value = [[6.5] [7.0] [7.8]]
squared_error = 0.0 samples = 1 value = [[4.57] [4.94] [5.48]]
squared_error = 0.0 samples = 1 value = [[4.63] [5.97] [6.65]]
water (kg/m3) ≤ 0.515 squared_error = 0.177 samples = 4 value = [[7.01] [7.4] [6.6]]
squared_error = -0.0 samples = 1 value = [[6.4] [6.5] [6.1]]
squared_error = 0.0 samples = 1 value = [[5.5] [6.8] [5.25]]
squared_error = 0.0 samples = 1 value = [[5.8] [6.3] [5.9]]
Amount(kg/m3) ≤ 0.044 squared_error = 0.079 samples = 3 value = [[6.847] [7.367] [6.3]]
squared_error = -0.0 samples = 1 value = [[7.5] [7.5] [7.5]]
water (kg/m3) ≤ 0.18 squared_error = 0.018 samples = 2 value = [[7.1] [7.55] [6.35]]
squared_error = 0.0 samples = 1 value = [[6.34] [7.0] [6.2]]
squared_error = 0.0 samples = 1 value = [[7.0] [7.4] [6.2]]
squared_error = 0.0 samples = 1 value = [[7.2] [7.7] [6.5]]

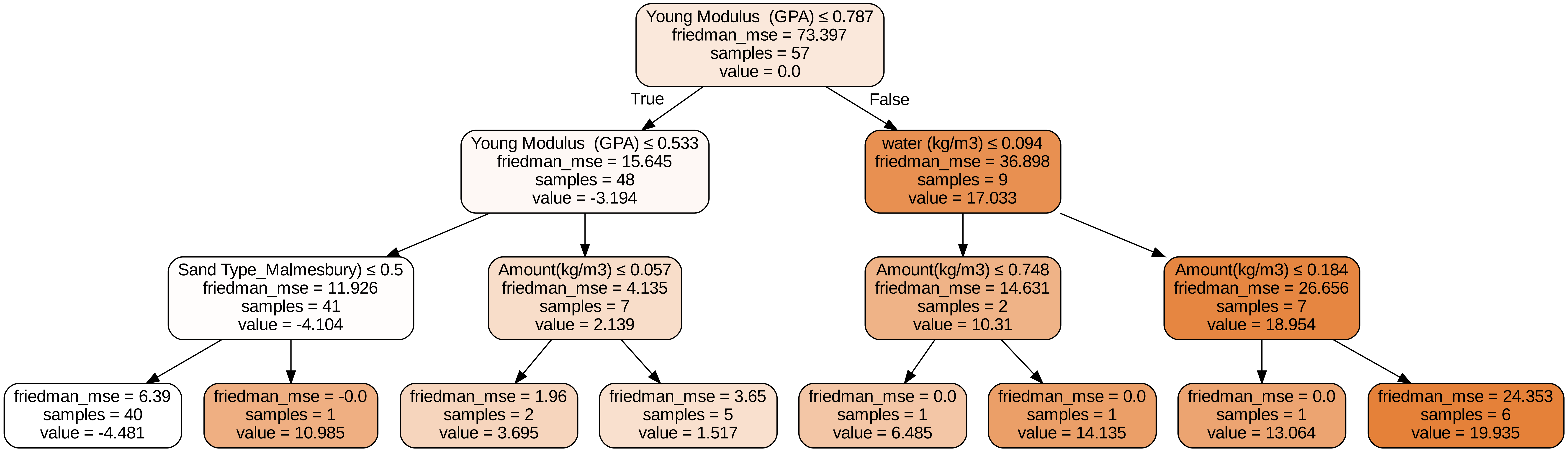
Young Modulus (GPA) ≤ 0.787
friedman_mse = 73.397
samples = 57
value = 0.0
True
False
Young Modulus (GPA) ≤ 0.533
friedman_mse = 15.645
samples = 48
value = -3.194
water (kg/m3) ≤ 0.094
friedman_mse = 36.898
samples = 9
value = 17.033
Sand Type_Malmesbury) ≤ 0.5
friedman_mse = 11.926
samples = 41
value = -4.104
Amount(kg/m3) ≤ 0.057
friedman_mse = 4.135
samples = 7
value = 2.139
Amount(kg/m3) ≤ 0.748
friedman_mse = 14.631
samples = 2
value = 10.31
Amount(kg/m3) ≤ 0.184
friedman_mse = 26.656
samples = 7
value = 18.954
friedman_mse = 6.39
samples = 40
value = -4.481
friedman_mse = -0.0
samples = 1
value = 10.985
friedman_mse = 1.96
samples = 2
value = 3.695
friedman_mse = 3.65
samples = 5
value = 1.517
friedman_mse = 0.0
samples = 1
value = 6.485
friedman_mse = 0.0
samples = 1
value = 14.135
friedman_mse = 0.0
samples = 1
value = 13.064
friedman_mse = 24.353
samples = 6
value = 19.935

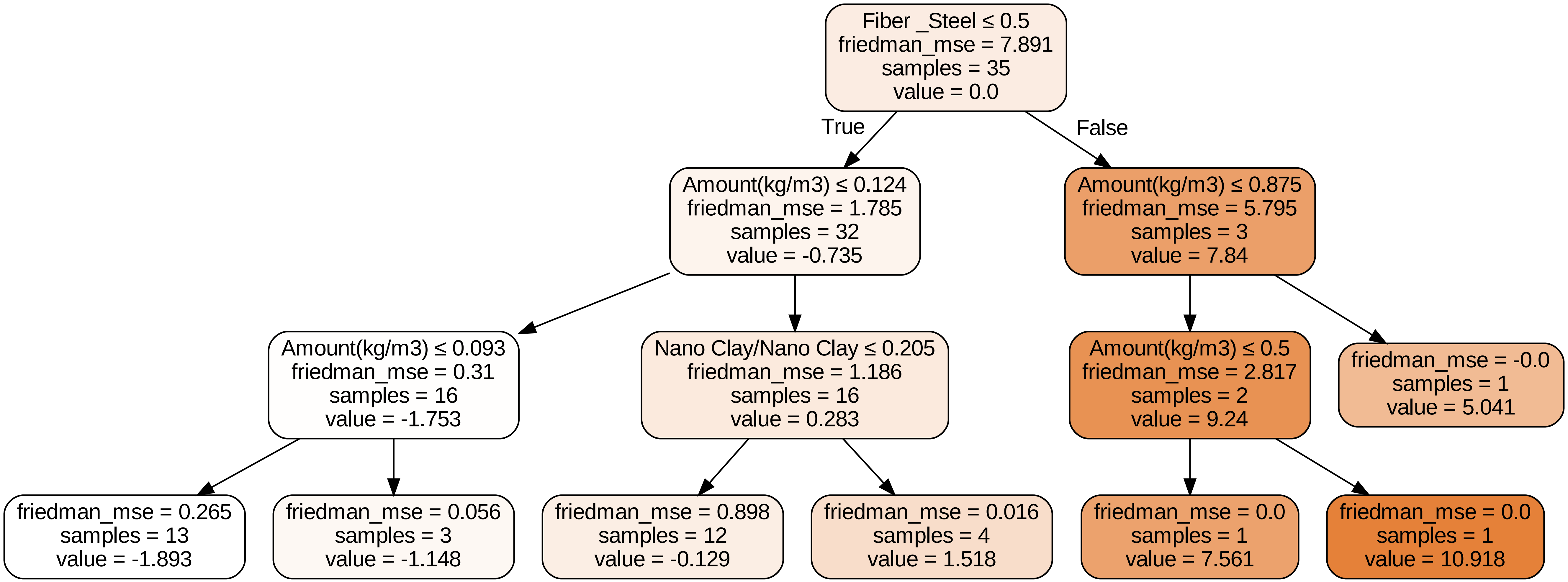
Fiber _Steel ≤ 0.5
friedman_mse = 7.891
samples = 35
value = 0.0
True
False
Amount(kg/m3) ≤ 0.124
friedman_mse = 1.785
samples = 32
value = -0.735
Amount(kg/m3) ≤ 0.875
friedman_mse = 5.795
samples = 3
value = 7.84
Amount(kg/m3) ≤ 0.093
friedman_mse = 0.31
samples = 16
value = -1.753
Nano Clay/Nano Clay ≤ 0.205
friedman_mse = 1.186
samples = 16
value = 0.283
Amount(kg/m3) ≤ 0.5
friedman_mse = 2.817
samples = 2
value = 9.24
friedman_mse = -0.0
samples = 1
value = 5.041
friedman_mse = 0.265
samples = 13
value = -1.893
friedman_mse = 0.056
samples = 3
value = -1.148
friedman_mse = 0.898
samples = 12
value = -0.129
friedman_mse = 0.016
samples = 4
value = 1.518
friedman_mse = 0.0
samples = 1
value = 7.561
friedman_mse = 0.0
samples = 1
value = 10.918

DOI	LINK
https://doi.org/10.1016/j.jobe.2022.104745	https://www.sciencedirect.com/science/article/abs
https://doi.org/10.1016/j.cemconres.2021.1063	https://www.sciencedirect.com/science/article/abs
https://doi.org/10.1016/j.matdes.2019.108088	https://www.sciencedirect.com/science/article/pii/
https://doi.org/10.1016/j.conbuildmat.2021.12	https://www.sciencedirect.com/science/article/abs
https://doi.org/10.1016/j.jobe.2021.102944	https://www.sciencedirect.com/science/article/abs
https://doi.org/10.1016/j.acme.2017.02.008	https://www.sciencedirect.com/science/article/abs
https://doi.org/10.1016/j.compstruct.2020.112	https://www.sciencedirect.com/science/article/abs
https://doi.org/10.1016/j.ceramint.2021.06.124	https://www.sciencedirect.com/science/article/abs
https://doi.org/10.1016/j.cemconres.2021.1063	https://www.sciencedirect.com/science/article/abs
https://doi.org/10.1016/j.conbuildmat.2019.11	https://www.sciencedirect.com/science/article/abs
https://doi.org/10.1007/978-3-030-49916-7_53	https://link.springer.com/chapter/10.1007/978-3-0
https://doi.org/10.1016/j.jclepro.2021.128720	https://www.sciencedirect.com/science/article/abs
https://doi.org/10.1016/j.matdes.2019.108088	https://www.sciencedirect.com/science/article/pii/
https://doi.org/10.1016/j.conbuildmat.2019.01.	https://www.sciencedirect.com/science/article/abs
https://doi.org/10.1016/j.conbuildmat.2017.12.	https://www.sciencedirect.com/science/article/abs
doi:10.3151/jact.19.1264doi:10.3151/jact.19.1264doi:10.3151/jact.19.1264	
https://doi.org/10.1016/j.cemconcomp.2021.10	https://www.sciencedirect.com/science/article/abs
https://doi.org/10.3390/ma11122352	https://www.mdpi.com/1996-1944/11/12/2352
https://doi.org/10.1016/j.conbuildmat.2019.01.	https://www.sciencedirect.com/science/article/abs
https://doi.org/10.1016/j.cemconcomp.2021.10	https://www.sciencedirect.com/science/article/abs
https://doi.org/10.1016/j.cemconres.2022.1068	https://www.sciencedirect.com/science/article/abs
https://doi.org/10.1016/j.conbuildmat.2021.12	https://www.sciencedirect.com/science/article/abs
https://doi.org/10.1016/j.conbuildmat.2020.11	https://www.sciencedirect.com/science/article/abs
https://doi.org/10.1016/j.conbuildmat.2021.12	https://www.sciencedirect.com/science/article/abs
https://doi.org/10.1016/j.cemconres.2021.1063	https://www.sciencedirect.com/science/article/abs
